# Organic coating on biochar explains its nutrient retention and stimulation of soil fertility

**DOI:** 10.1038/s41467-017-01123-0

**Published:** 2017-10-20

**Authors:** Nikolas Hagemann, Stephen Joseph, Hans-Peter Schmidt, Claudia I. Kammann, Johannes Harter, Thomas Borch, Robert B. Young, Krisztina Varga, Sarasadat Taherymoosavi, K. Wade Elliott, Amy McKenna, Mihaela Albu, Claudia Mayrhofer, Martin Obst, Pellegrino Conte, Alba Dieguez-Alonso, Silvia Orsetti, Edisson Subdiaga, Sebastian Behrens, Andreas Kappler

**Affiliations:** 1 Geomicrobiology, Center for Applied Geosciences, University of Tuebingen, Sigwartstrasse 10, Tuebingen, 72076 Germany; 2 School of Environmental and Life Sciences, Chemistry, University of Newcastle, Callaghan, NSW 2308 Australia; 3 School of Materials Science and Engineering, University of New South Wales, Kensington, NSW 2052 Australia; 4 Nanjing Agricultural University, Nanjing, 210095 China; 5 Ithaka Institute for Carbon Strategies, Ancienne Eglise 9, Arbaz, 1974 Switzerland; 6 Department of Soil Science and Plant Nutrition, WG Climate Change Research for Special Crops, Hochschule Geisenheim University, von-Lade Str. 1, Geisenheim, 65366 Germany; 7 Department of Soil and Crop Sciences and Department of Chemistry, Colorado State University, Fort Collins, CO 80523 USA; 8 Department of Molecular, Cellular, and Biomedical Sciences, University of New Hampshire, Durham, NH 03824 USA; 9 National High Magnetic Field Laboratory, Florida State University, 1800 East Paul Dirac Drive, Tallahassee, FL 32310-4005 USA; 10 Austrian Cooperative Research, Centre for Electron Microscopy and Nanoanalysis, Steyrergasse 17, Graz, 8010 Austria; 11 BayCEER Analytics, University of Bayreuth, Bayreuth, 95440 Germany; 12 Dipartimento di Scienze Agrarie e Forestali, Università degli Studi di Palermo, v.le delle Scienze ed. 4, Palermo, 90128 Italy; 13 Institute of Energy Engineering, Chair for Energy Process Engineering and Conversion Technologies for Renewable Energies, Technische Universität Berlin, Fasanenstraße 89, Berlin, 10623 Germany; 14 Environmental Mineralogy and Chemistry, Center for Applied Geoscience, University of Tuebingen, Sigwartstrasse 10, Tuebingen, 72076 Germany; 15 Department for Civil, Environmental, and Geo-Engineering, University of Minnesota, 500 Pillsbury Drive S.E, Minneapolis, MN 55455-0116 USA; 16 BioTechonology Institute, 140 Gortner Labs, 1479 Gortner Avenue, St. Paul, MN 55108-6106 USA; 17 Present Address: Environmental Analytics, Agroscope, Reckenholzstraße 191, 8046 Zurich, Switzerland

## Abstract

Amending soil with biochar (pyrolized biomass) is suggested as a globally applicable approach to address climate change and soil degradation by carbon sequestration, reducing soil-borne greenhouse-gas emissions and increasing soil nutrient retention. Biochar was shown to promote plant growth, especially when combined with nutrient-rich organic matter, e.g., co-composted biochar. Plant growth promotion was explained by slow release of nutrients, although a mechanistic understanding of nutrient storage in biochar is missing. Here we identify a complex, nutrient-rich organic coating on co-composted biochar that covers the outer and inner (pore) surfaces of biochar particles using high-resolution spectro(micro)scopy and mass spectrometry. Fast field cycling nuclear magnetic resonance, electrochemical analysis and gas adsorption demonstrated that this coating adds hydrophilicity, redox-active moieties, and additional mesoporosity, which strengthens biochar-water interactions and thus enhances nutrient retention. This implies that the functioning of biochar in soil is determined by the formation of an organic coating, rather than biochar surface oxidation, as previously suggested.

## Abstract (web summary)

Biochar promotes plant growth via a slow release of nutrients; however, a mechanistic understanding of nutrient storage in biochar is lacking. Here, using high-resolution spectromicroscopy and mass spectrometry, the authors identify an organic coating on co-composted particles that enhances nutrient retention.

## Introduction

Biochar is the product of O_2_-limited thermal treatment of biomass (pyrolysis) and is used in agriculture as a livestock feed supplement, compost additive and soil amendment as well as for manure treatment^1^. It is applied to improve animal, plant and soil health, to reduce nutrient losses by volatilization or leaching, to prevent soil erosion, and to improve soil water retention, soil carbon content and the long-term fertility of agricultural soils^2, 3^. Biochar is very recalcitrant and can contribute to climate change mitigation by the sequestration of stable carbon^4^ and reduction of agricultural emissions of CO_2_, N_2_O and CH_4_^5^.

Positive impacts of biochar on (agro)ecosystems are often explained by the porosity and sorption capacity^3^, redox properties^6, 7^, and liming capacity of biochar^8^ and by biochar's influence on soil structure^9, 10^, water holding capacity^11^ and nutrient transformations in soil^2^. However, despite these insights into biochar functioning in soil, a detailed mechanistic understanding of how biochar influences plant growth is still lacking. Biochar was shown to increase crop yield by up to 400% compared to fully fertilized controls^12^, while the overall mean of biochar-induced yield increases is 18% according to meta-analyses^13^. However, many studies used high biochar application amounts of >10 t ha^−1^, which is not economically feasible. Recent research suggested that biochar should be combined with organic amendments to increase soil fertility even when biochar is applied at low (0.5–2 t ha^−1^) biochar application rates^14, 15^. Co-composting, which consists of mixing biochar with manure or other compost feedstock with high contents of both nutrients and labile organic carbon before starting an aerobic composting process, was shown to enhance the agronomic performance of biochar as a soil amendment^16^. The co-composted biochar handpicked from the final biochar-amended compost was shown to promote plant growth beyond the combination of pristine biochar with either mineral fertilizer or mature non-biochar-amended compost. This phenomenon was explained by co-composted biochar’s slow release of essential plant nutrients like nitrate and phosphate^16^. However, despite evidence for surface oxidation, sorption of organic molecules and increase in carboxyl groups on biochar surfaces^17, 18^, the underlying mechanisms and the impact of co-composting on the biochar microstructure remain unclear. A mechanistic understanding of how co-composting impacts biochar’s reactivity and function is a prerequisite for large-scale and global beneficial use of biochar in agriculture, additionally for understanding the formation of extremely fertile Amazonian and African Dark Earths that received anthropogenic input of both nutrient-rich organic matter and pyrogenic carbon centuries ago^19, 20^, and eventually for the development of low-cost biochar-based fertilizers that can promote high crop yields with comparably small application doses of biochar^21, 22^. In addition, mechanistic insights into biochar co-composting will help to improve our current understanding of how biochar ages in soil^23^.

On the surface of co-composted biochar, we identified an organic coating by applying a suite of spectro(micro)scopical techniques and argue that this coating controls the interaction of co-composted biochar with water and nutrients. We found that a similar coating was present in non-composted but soil-aged biochar. This study demonstrates why co-composted or organically amended biochar helps optimize its agronomic performance.

## Results

### Extraordinary nutrient retention by co-composted biochar

**Fig. 1:**
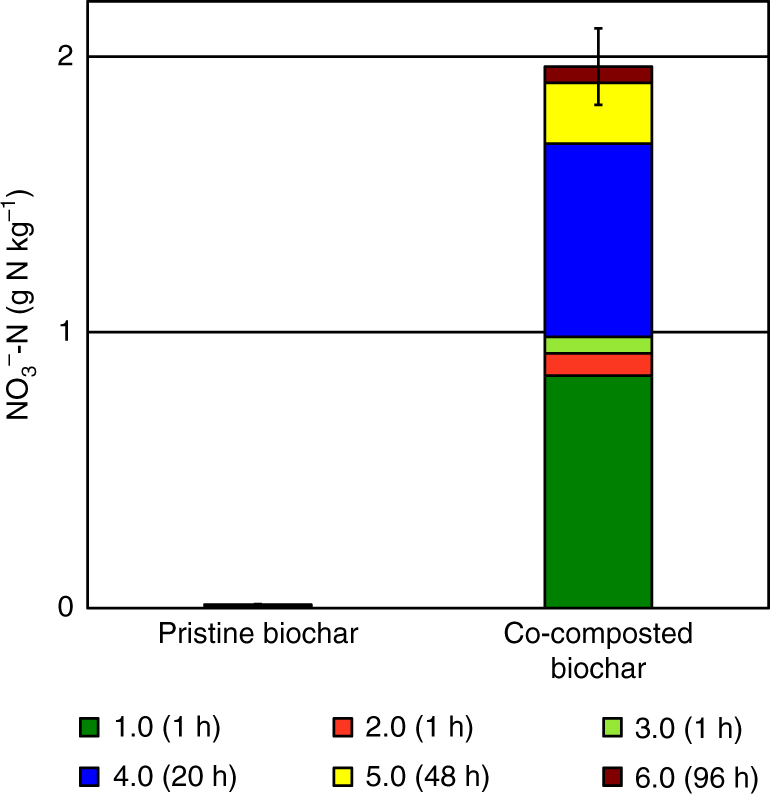
Biochar nitrate content based on repeated extractions with 2 M KCl. Each segment of the stacked bar represents one step of six consecutive extractions with KCl: the first, second and third extraction took 1 h each, the fourth took 20 h, the fifth 48 h, and the sixth took 95 h. Error bar represents 1 S.E. of the total extraction carried out in triplicates

We manually picked co-composted biochar particles (BC_comp_) after 60 days of aerobic composting plus 6 months of storage and analyzed them without any further sample preparation^24^. BC_comp_ contained 2.0 ± 0.1 g NO_3_^−^-N kg^−1^, of which only 43% could be extracted by conventional extraction (Fig. 1), clearly demonstrating the potential of co-composted biochar as a slow release fertilizer. We investigated both pristine (fresh, no post-production treatment, no detectable nitrate) biochar (BC_prist_) and BC_comp_ with a suite of spectro(micro)scopic and mass-spectrometric techniques to identify the underlying molecular mechanism(s) for the remarkable retention of anionic and cationic nutrients by co-composted biochar^16^ and to evaluate the impact of co-composting on the long-term stability of biochar, i.e., its carbon sequestration potential.

### Spectromicroscopy and mass spectrometry

**Fig. 2:**
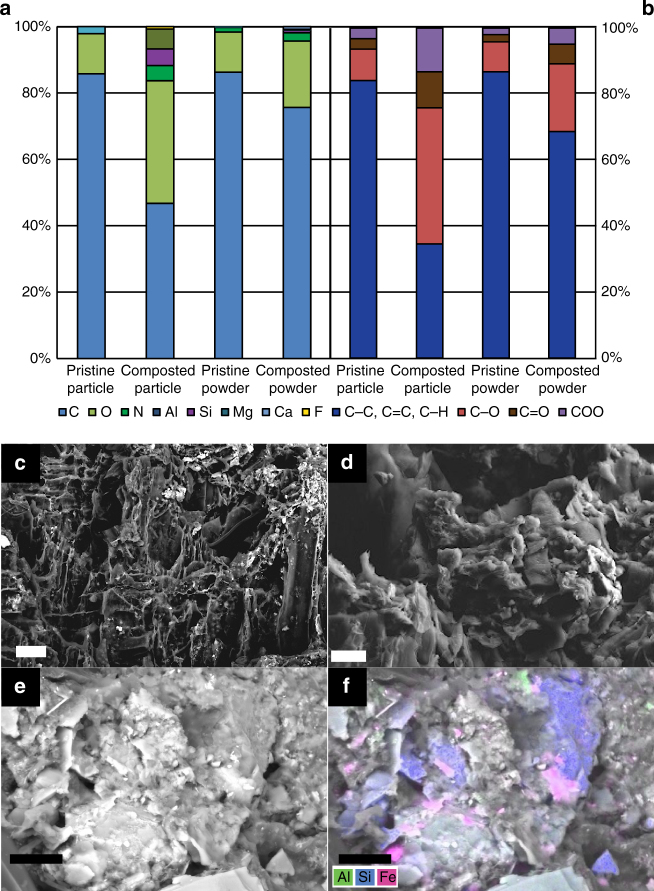
Identification of biochar surface modifications induced by co-composting. **a** Elemental composition of the surface (particle) and the bulk (powder) biochar according to X-ray photoelectron spectroscopy (XPS). **b** Carbon-containing functional groups according to region scans (higher energy resolution) of the same samples. **c**–**f** Scanning electron micrographs of co-composted biochar with **c** a region with degraded, but still exposed original biochar surfaces showing typical porous structure (scale bar, 20 µm); **d** a region of biochar surface partially covered with organic coating (scale bar, 10 µm); **e** a region of biochar surface completely covered with organic coating (scale bar, 600 µm). **f** map overlay of **e** showing the heterogeneous distribution of Al (green), Si (blue) and Fe (red) within the organic coating as quantified by energy-dispersive X-ray spectroscopy

First, we used^13^C solid-state nuclear magnetic resonance (NMR) spectroscopy, X-ray photoelectron spectroscopy (XPS) and scanning electron microscopy (SEM) to identify co-composting-induced changes in biochar carbon speciation on the bulk level and structural and chemical changes of the biochar surface. NMR spectroscopy did not reveal significant differences in C speciation between BC_prist_ and BC_comp_ on the bulk level (Supplementary Fig. 1). In line with this, bulk XPS, i.e., XPS of biochar particles powdered and sieved to 100 µm, only showed a minor increase in oxidized carbon species (C–O, C = O, COO, Fig. 2a-b). However, XPS applied on whole particles revealed a considerable increase in the O/C ratio and increase in N content by co-composting (Fig. 2a-b). Since XPS is a surface sensitive technique that provides information on the outermost ~10 nm this also suggests that only the biochar surface, not the bulk biochar particles, is altered during co-composting. SEM revealed that this surface alteration of BC_comp_ is not homogeneous, but that there were regions of degraded biochar pores (Fig. 2c) and hotspots of partially and completely coated biochar surfaces (Fig. 2d, e). Elemental mapping using energy dispersive X-ray spectroscopy (EDS) indicated heterogeneous contents of Fe, Al and Si in the coating (Fig. 2f).

**Fig. 3:**
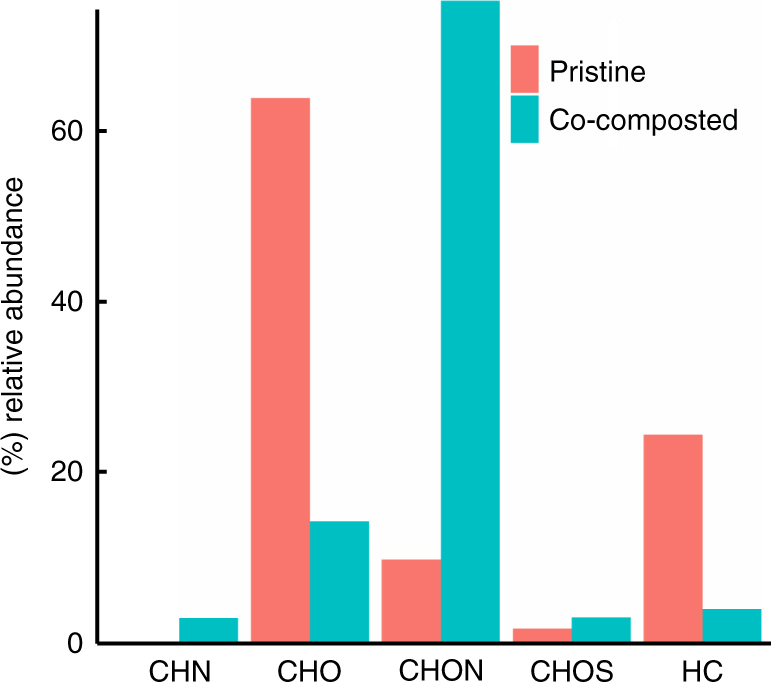
In situ characterization of the organic coating by DAPPI FT-ICR MS. Relative abundance of C, H, O, N and S bearing compound classes on the surface of pristine and co-composted biochar according to desorption atmospheric pressure photoionization Fourier-transformed ion-cyclotron resonance mass spectrometry (DAPPI FT-ICR MS)

**Fig. 4:**
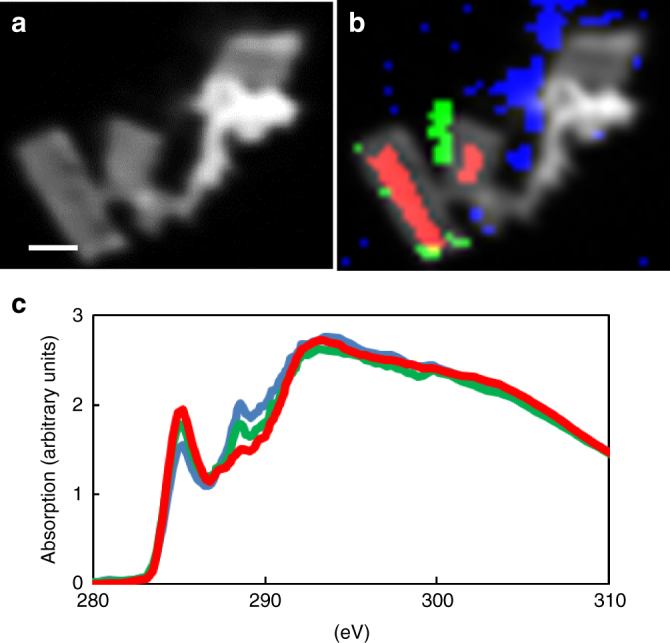
Scanning transmission X-ray microscopy of co-composted biochar. **a** Average image on linear absorbance scale (optical density) calculated from a stack across the C1s absorption edge (278–330 eV) of an ultra-thin section of co-composted biochar. Scale bar, 500 nm. **b** C1s stack with overlay of regions of interest for downstream data evaluation: green—regions on or directly adjacent to the two biochar particles with relatively high N/C ratio (based on X-ray absorption ratio), red—manually defined region of the particle center, blue—all regions in the analyzed area with high N/C ratio, includes the green marked region. Same scale as **a**. **c** X-ray absorption spectra extracted from the regions defined in **b** with the respective colors, the blue spectrum again includes the green region. Spectra reveal a relative increase in absorption at the 1s-pi* transition (288.6 eV; R-COOH; CO^62^) from particle center (red) to particle associated high N/C (green) to all areas with high N/C (blue). In-depth analysis of this and two other regions of interest is displayed in Supplementary Fig. 3B, C. Comparison of with pristine biochar is displayed in Supplementary Fig. 3A

We characterized the coating in situ with mass spectrometry and spectroscopic techniques across a range of both spatial and energy resolutions to identify the chemical and physical properties of the surface modification. Desorption atmospheric pressure photoionization Fourier-transformed ion-cyclotron resonance mass spectrometry (DAPPI FT-ICR MS) applied to biochar particles provides precise information on the elemental composition of compounds available for desorption on the biochar surface. According to DAPPI FT-ICR MS, co-composting increased the relative abundance of CHON (C_W_H_X_O_y_N_z_) class compounds and decreased the relative abundance of CHO (C_x_H_y_O_z_) class compounds (Fig. 3). Furthermore, co-composting increased the abundance-weighted N/C ratio of all formulas assigned to the compounds that desorbed from the biochar surface at atmospheric pressure from 0.008 to 0.029, and increased the abundance-weighted O/C ratio of such formulas from 0.388 to 0.618. Within the CHON class, the van Krevelen diagram (Supplementary Fig. 2) suggests an increase in the O/C ratio of the desorbed compounds, consistent with an actual increase in the abundance-weighted O/C ratio from 0.278 to 0.566. To accurately identify carbon functional groups and spatial distribution of N in ultra-thin sections of BC_comp_, we used scanning transmission X-ray microscopy (STXM) which has a spot size of 30–50 nm. We could clearly demonstrate the heterogeneous distribution of nitrogen suggesting that there are hotspots of nutrient retention on the outer surface and inside pores of the biochar, rather than a homogenous enrichment and these hotspots showed a higher absorption by carboxylic moieties (Fig. 4). However, beyond this change, there are neither considerable differences in carbon speciation between bulk co-composted biochar and these hotspots nor between pristine and co-composted biochar (Supplementary Fig. 3).

**Fig. 5:**
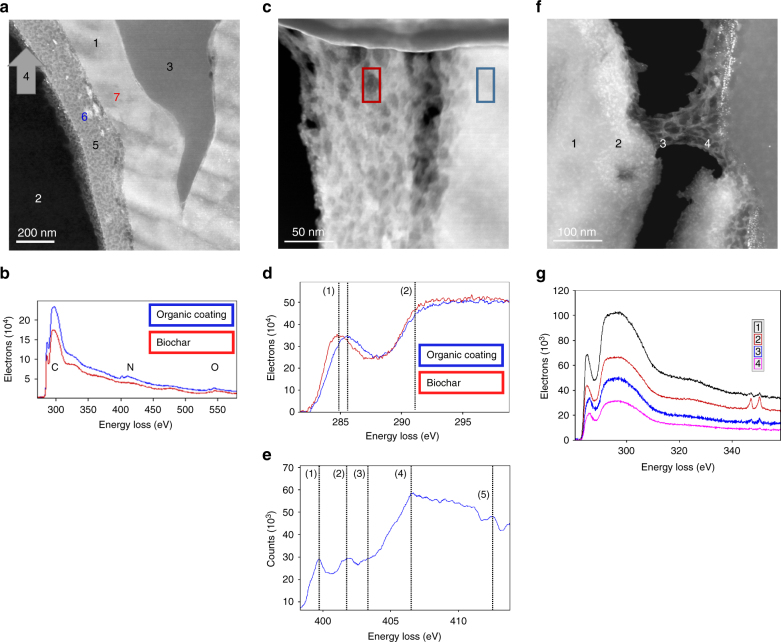
Scanning transmission electron microscopy and electron energy loss spectroscopy of organic coating on co-composted biochar. Micrographs were obtained from different ultra-thin sections of the same biochar particle. **a**, **b** STEM HAADF micrograph and EELS spectra of an ultra-thin section of co-composted biochar revealing an organic coating. Position 1: biochar with N and O below detection limit of EELS; 2: epoxy resin; 3: empty hole (resin did not penetrate this pore); 4: gold that was sputtered onto the biochar particle before embedding to identify the sample surface; 5: organic coating, porous appearance, contains N and O according to EELS spectra shown in **b**; 6 and 7: Location of EELS sum spectra shown in **b**. **b** EELS spectra revealing only minor differences in C speciation between biochar (red) and organic coating (blue), but considerably increased content of N in the coating. Letters indicate absorption edge (K shell) of C, N and O. **c** Closeup on a region of thick (~120 nm) organic coating, probably located inside a biochar pore (no gold coating detectable). **d** C-K near edge EELS spectrum of biochar matrix and coating as marked in **c** showing a peak shift of the C = C 1s–π* transition (1) of aromatic carbon by ~0.5 eV (284.9–285.5 eV). C–C 2s-2pz σ* transition (2) was not altered (291.2 eV). **e** N-K near edge EELS spectrum of coating as marked in **c** showing (1) imine N 1s–π* transition at 399–400 eV; (2) amide N 1s–3p/π* transition at 401.3 eV; (3) nitro N 1s–π* transition at 403.6 eV; (4) corresponds to HC ≡ N* transition at 406.8 eV with σ* resonance position at 420.8 eV^63^. **f** Organic coating formed at a biochar surface that is rich in Ca hotspots of bright appearance in the HAADF micrograph. Coating is shearing off as an artefact of the mechanical force applied during sample preparation, most likely by the microtome, which indicates a rather plastic nature of the coating compared to the biochar that broke (lower part of the micrograph). Thickness of coating varies between ~20 and 50 nm and the presence of gold indicates a semi-exposed position in the original biochar particle. **g** EELS spectra from regions indicated in **f** showing the peak shift of the C = C 1s–π* transition and the presence of Ca in the region of the bright spot in the outermost region of the biochar

Scanning transmission electron microscopy (STEM) equipped with electron energy loss spectroscopy (EELS) allows sub-micron scale resolutions on ultra-thin sections of the biochars and information on the carbon and nitrogen speciation in precisely (nm-range) defined regions. With STEM, we identified a meso-porous organic coating of up to 200 nm thickness on a substantial share of the investigated surfaces of BC_comp_ (Fig. 5 and Supplementary Fig. 4). STEM samples were sputter-coated with gold on a three-dimensional (3D) moving sample holder prior to sample preparation to clearly differentiate the sample-epoxy resin border. The organic coating was thicker on pore surfaces than on exposed, outer surfaces, where it was mostly limited to 10–20 nm non-porous spots (Supplementary Fig. 4D). The coating was completely absent in BC_prist_ (Supplementary Fig. 5). EELS revealed a higher N content of the coating compared to the carbonaceous biochar matrix and minor alterations of the C speciation (Fig. 5b–e) including peak shifts of the aromatic C = C 1s–π* transition by 0.5 eV from co-composted biochar matrix to the coating (Fig. 5c, d). Additionally, we found nanometer-sized hot-spots of silica and other elements in both pristine and co-composted biochar (Supplementary Fig. 5B). Similar hotspots of calcium were identified on the surface of the co-composted biochar, right under the coating (Fig. 5f, g). A similar phenomenon was described for pyrogenic organic matter isolated from Terra Preta^25^.

Additional STEM on crushed BC_comp_ revealed the formation of complex organo-mineral associations involving Ca and redox-active Fe (Supplementary Fig. 6). Similar structures have been suggested as hotspots for redox reactions in the liquid and gaseous phase of soils and suggest that the organic coating is not only a porous matrix for nutrient retention, but also a potential location for nutrient transformations^26, 27^.

**Fig. 6:**
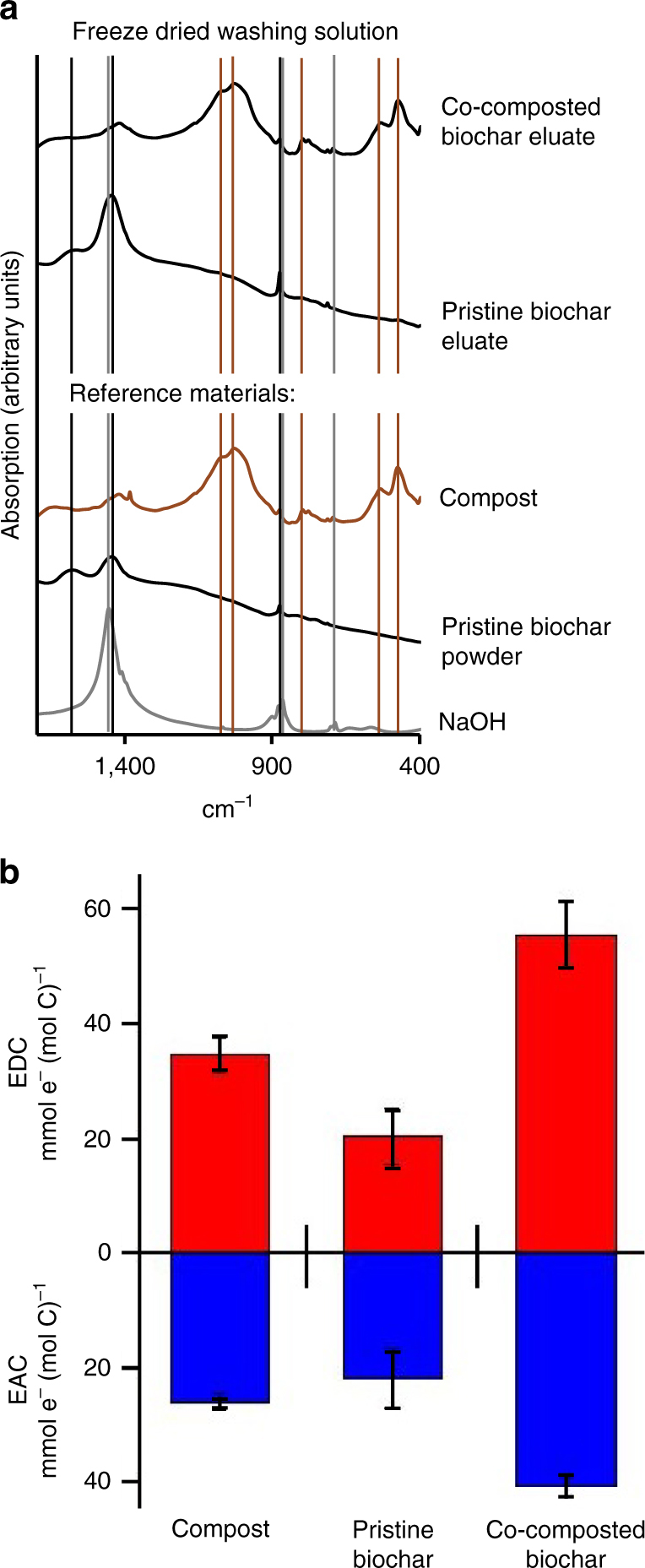
Ex situ analysis of the organic coating extracted from the biochar with 0.05 M NaOH. **a** Fourier-transformed infrared (FTIR) spectra of freeze-dried eluates of pristine and co-composted biochar. FTIR spectra of pure compost, pristine biochar and NaOH were measured as a reference. **b** Electron accepting (blue) and electron donating (red) capacity (EAC and EDC) of biochar eluates normalized to their carbon content. Error bars represent one standard deviation of at least five replicates

To obtain more detailed information on the identity and properties of this organic coating, the material was characterized ex situ by first pre-washing the BC_prist_ and BC_comp_ with water to remove loosely attached compost organic matter followed by extraction of the biochar particles with 0.05 M NaOH^28^ to isolate the organic coating for further analysis after filtration to <0.45 µm. After this procedure, the co-composted biochar lost its brownish color and is almost completely black again (Supplementary Fig. 7). Ion chromatography and elemental analysis of the extracted coating revealed an increased content of N and C, especially nitrate, organic carbon, carbonate, Ca and K in BC_comp_ compared to extracts obtained from BC_prist_ (Supplementary Table 4). The^1^H solution NMR spectrum of the BC_prist_ extract comprised only few defined peaks, while the spectrum of BC_comp_ extracts was dominated by broad peaks that did not allow detailed evaluation (Supplementary Fig. 10). The complexity of the spectrum showed that the formation of the organic coating in the co-composted biochar cannot solely be explained by oxidation of the original biochar carbon but entirely new species/entities of carbon were evidently introduced into the biochar porous system during co-composting, which is supported by data obtained by spectrofluorometry (Supplementary Fig. 11). This compost derived organic carbon have had to be sorbed on the biochar because if it just had been weakly associated with the biochar, it would have been removed already by the first washing step (10 min with water), while analysis was performed on the eluate of the third washing step. In line with that, Fourier-transformed infrared (FTIR) spectroscopy of freeze-dried BC_comp_ extracts revealed similar bands as found in both a powdered BC_prist_ and powdered compost reference (Fig. 6a). This suggests that biochar nano-particles were associated with compost organic matter to form the porous coating. Biochar nano-particles can form during co-composting by physical disintegration, potentially by graphitic sheet expansion, when biochar sorbs water from the compost^29^. Quantification of the electron exchange capacity (EEC), i.e., the sum of electron accepting and donating capacity^6^, revealed that the EEC per mmol of carbon of BC_comp_ eluates is higher than the EEC both of BC_prist_ and of compost eluates (Fig. 6b). Thus, the chemistry of the coating is different from both of these compounds. Our data suggests that the reaction of compost organic matter with biochar created more reactive carbon moieties that then formed the organic coating. Although, looking at EDC/EEC ratios, eluate of co-composted biochar presents similar ratio than compost, indicating that the co-composted biochar presents the same redox state than the compost. From an absolute point of view, pristine biochar shows lower EEC which is increased by co-composting which could be attributed to conformational re-arrangements of carbon moieties taking place during the sorption compost organic matter onto pristine biochar, making the redox-active moieties more exposed to the electrochemical techniques. Alternatively, biochar might have preferentially sorbed compounds with a higher EEC than the average EEC of the compost. However, there was no clear evidence of fractionation of the organic matter as consequence of the sorption, as suggested by fluorescence emission spectroscopy (Supplementary Fig. 11).

### Porosity and interaction with water

**Fig. 7:**
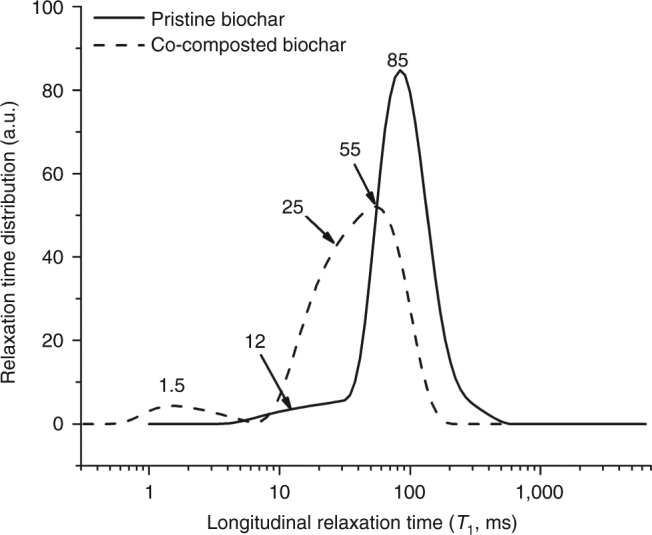
Analysis of biochar surface hydrophilicity by fast field cycling nuclear magnetic resonance relaxometry: Relaxograms, i.e., distribution of relaxation times, of pristine and co-composted biochar. The shorter the *T*_1_ value, the better water molecules are anchored to the biochar surface. Conversely, the longer the longitudinal relaxation time, the weaker are the interactions between water and the surface of the biochar system

**Fig. 8:**
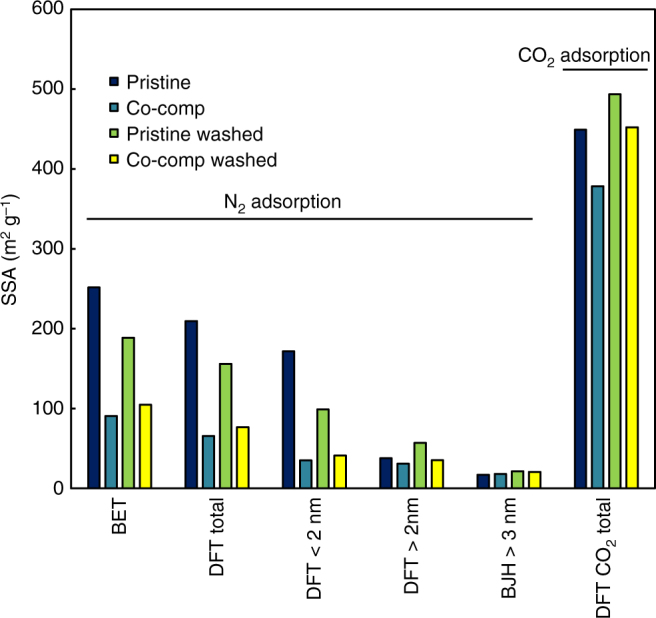
Specific surface area (SSA) according to N_2_ and CO_2_ gas adsorption. Total SSA according to the Brunauer, Emmett and Teller method, total SSA according to the quenched-solid density functional theory (QSDFT) method, SSA due to micropores according to the QSDFT method, SSA for mesopores up to 34 nm according to the QSDFT method, SSA for pores bigger than 3 nm according to the Barrett, Joyner and Halenda method and SSA of micropores with CO_2_ adsorption and applying the non local density function theory method of pristine and co-composted biochar, pristine biochar after washing and co-composted biochar after washing

To identify the mechanism responsible for the retention of polar nutrients such as nitrate by the observed porous organic coating of biochar surfaces, we used fast field cycling NMR (FFC NMR) relaxometry quantifying the strength of biochar interactions with water^30^. We found that co-composting changed the distribution of relaxation times (Fig. 7 and Supplementary Fig. 12), i.e., time needed for protons to recover the longitudinal component after magnetization^31^. The different relaxation times indicate the strengths of interactions of water molecules with biochar pore surface as well as with pore water-dissolved or suspended compounds^30, 32^. Lower relaxation times in BC_comp_ suggest the presence of non-homogeneously distributed H-bond donor/acceptor groups. Thus, water is better anchored to the surfaces of BC_comp_ and thus less mobile, which can explain the retention of water-dissolved nutrients, such as nitrate. Liquid chromatography organic carbon detection (LC-OCD, Supplementary Tables 5–7, Supplementary Fig. 17 and Supplementary Discussion) of aqueous biochar extracts revealed a high content of humic-substance-like organic molecules, that might act as surfactants that facilitate this process. In addition, the organic coating can shrink pore sizes thereby reducing water mobility^31^. This is supported by gas adsorption measurements that showed that co-composting led to ~18% reduction in specific surface area (SSA) according to CO_2_ adsorption, especially in the pore size range of 0.65–0.85 nm, and ~64% SSA reduction to N_2_ adsorption, especially in the pore size range of 1.25–1.45 nm (Fig. 8, Supplementary Fig. 13 and Supplementary Discussion). Thus, among micropores, the larger, more accessible pores were preferentially clogged. Pores > 3 nm were not affected; however, pore clogging might be underestimated due to new mesopores of the organic coating increasing total porosity. In essence, CO_2_ gas adsorption reveals that the reduction of SSA by composting is less severe than reported previously^33^.

Collectively, our data showed that co-composting results in the formation of a heterogeneous, meso-porous and hydrophilic organic coating on biochar surfaces including inner pore surfaces. The coating was identified as a composite of the original compost organic matter and biochar nano-particles and it is enriched in N (especially NO_3_^−^), K, Ca, Si, Fe and Al. It has a higher N/C ratio than the biochar matrix, a higher redox activity, and adds another porous component to the porous biochar. However, bulk carbon speciation of the biochar were not altered by co-composting. This suggests that co-composting of biochar does not affect its long-term stability in soil, however, this needs further evidence from incubation studies.

Our data provide, for the first time, mechanistic evidence on a molecular scale as to why the combination of biochar and organic amendments yields optimal agronomic benefits by biochar amendment. The enhanced interaction of co-composted biochar with water (hydrophilicity of organic coating decreases biochar’s hydrophobicity) is most likely responsible for its improved nutrient retention and therefore its superior agronomic performance^16^. Co-composting of biochar with nutrient-rich feedstock or other ways of treating nutrient-rich waste streams with biochar (e.g., liquid manure) is therefore key to increase biochars’ agronomic value.

### Organic coating on soil-aged biochars

We additionally found that the organic coating is not restricted to the biochar example presented in this study. STEM also revealed an organic coating in another co-composted wood biochar (Supplementary Fig. 14) and in a soil-aged biochar (BC_soil_, Supplementary Fig. 15). Basic biochar properties of BC_soil_ were similar to those of BC_prist_ and BC_comp_ (similar feedstock, same process and pyrolysis conditions), but it was produced in 2012 and amended to a field experiment after soaking in a fertilizer solution. The organic coating on BC_soil_ has slightly different properties (lower electron accepting capacity, no evidence for incorporation of biochar nano-particles, different interaction with water, Supplementary Fig. 16) compared to BC_comp_. Nevertheless, the evidence of organic coatings on both the composted and the soil-aged biochar strongly suggests that the organic coating of natural and anthropogenic (biochar) pyrogenic carbon is likely a ubiquitous phenomenon and corresponds to the finding that biochar stabilizes rhizodeposits^34^. This could explain the previously reported nitrate-retention capacity of biochar particles aged for 2.5 years in a field experiment^35^. Hence it is reasonable to assume that similar coatings may also have formed both on anthropogenic pyrogenic carbon in Amazonian and African Dark Earths soils and as well on natural pyrogenic carbon in soils, which was recently shown to account for on average 13.7% of the global soil organic carbon pool^36^.

## Discussion

Our study revealed that the formation of an organic coating on biochar, that was observed on all biochar particles investigated in this study, is the dominant molecular mechanism of biochar alteration by co-composting and soil ageing. This mechanism replaces the paradigm of surface oxidation of native biochar carbon as the dominant process of ageing. It might partially explain biochar’s longevity in soil^4^, as the organic coating might protect aromatic biochar surfaces to some extent from further (oxidative) degradation. Surface oxidation might play a certain role, as biochar particles are not completely covered by the organic coating (Fig. 2c, d). Moreover, functionalization of biochar surfaces by oxidation might facilitate the formation of the organic coating, as oxidized carbon moieties facilitate the interaction of biochar with organic molecules by hydrogen bonds^18^. However, our study clearly shows that increased abundance of oxidized carbon moieties like carboxylic groups or increased O/C ratios of biochar should not be simply assigned to surface oxidation, when the data is obtained from bulk analysis.

Future research needs look at the mechanisms of the formation of the organic coating. Although we presented evidence through LC-OCD that the organic coating consists of humic-like substances, microbial activity could play an important role in the formation of the organic coating. It needs to be clarified if the increase in EEC is the result a chemical alteration of organic matter or the result of preferential sorption of organic matter with a high EEC.

We conclude that the combination of biochar with non-pyrogenic organic matter (e.g., manure) is the key strategy to develop carbon-fertilizer carriers that are effective at low application rates^21, 22^. Annual application of such biochar-based fertilizers could sequester carbon and mitigate global warming^5^—a global mitigation strategy that could be implemented due to its economic value for farmers, without the necessity for further subsidies.

## Methods

### Materials

Biochar was produced from mixed woody waste materials via slow pyrolysis (700 °C) by Swiss Biochar (Belmont-sur-Lausanne, VD, Switzerland) with a Pyreg reactor^37^ and was characterized by Eurofins Umwelt Ost GmbH, Halsbrücke-Tuttendorf, Germany, as requested by the European Biochar Certificate^1^. Results are displayed in Supplementary Tables 1–3. This biochar was part of the COST Action-TD1107 biochar ring trial^38^ and both pristine/non-co-composted and co-composted aliquots from the same composting experiment were subject to previous studies^39^.

Biochar was co-composted at the Ithaka Institute at St. Léonard, VS, Switzerland, from August to October 2014. Biochar was mixed into compost feedstock composed of cow, horse and poultry manure and green waste at a rate of 4.3% (dry matter w/w). Compost was managed aerobically as described by Kammann et al.^24^ resulting in temperatures above 60 °C for more than 2 weeks. Compost feedstock was composted without biochar amendment as a control. After frost-protected storage during winter, biochar was picked manually from the mature compost in spring 2015. For biochar picking, 50 kg of biochar-amended compost were stored for analysis in a plastic bag. For each analytical procedure (extraction, characterization), a subsample of compost was collected as a composite sample from this bag. 10–30 char particles were separated from this individual subsample and then prepared for analysis. The actual number of biochar particles finally involved in analysis varied strongly between the different techniques. E.g. only 3 particles of each treatment could by analyzed by STEM and 2 for STXM, while extraction or powder-based analysis (gas adsorption) where based on at least 20 particles.

The same type of biochar was already purchased from Swiss Biochar in 2012 (separate analysis in Supplementary Tables 1–3). It was soaked in a diluted commercial fertilizer solution overnight (1:1 w/v, 1.25 g N kg^−1^ biochar) and incorporated at a rate of 60 Mg ha^−1^ into the upper 15 cm of a Terric Anthrosol (top- and subsoil of a Cambisol mixed by construction activities) at the Tuebingen-Sand field site (lat. 48.5342, long. 9.0711). The plot was cropped with Emmer wheat (*Triticum dicoccon*) in 2012 and winter vetch (*Vicia villosa*) in 2013 and green fallow thereafter. Biochar was manually picked from soil samples obtained from the upper 15 cm of three sampling sites in spring 2015.

### Serial extraction of nitrate

Nitrate was repeatedly extracted from both pristine and co-composted biochar 1:10 (w/v) with 2 M KCl in 50 ml Falcon tubes (*n* = 3) on a roller shaker (ROLLER 10 digital, IKA, Staufen, Germany) at 50 r.p.m. at room temperature (22 ± 3 °C). After each extraction step, the extractant was decanted through a 0.5 mm sieve. The volume of the decanted extractant was measured to correct the data for the residual extractant (2 M KCl) that cannot be removed by decanting (residual = water content of biochar + added extractant—decanted extractant) which carries a small quantity of already extracted nitrate from one to the subsequent extraction. Fresh 2 M KCl was added to the biochar at the same volume. Each sample was extracted six times: three times for 1 h, for 18 h, for 48 h and for 96 h, respectively, resulting in a total extraction time of 167 ± 1 h. Nitrate was quantified using a continuous flow analyzer (SEAL Analytical, Germany) after reduction of the nitrate to nitrite with hydrazine, which was prepared according to SEAL’s advice for soil extracts. Nitrite was quantified by UV–vis absorption at 550 nm after reaction with N-1-naphtyl-ethylendiamin. The SEAL system is equipped with a dialysis membrane that removes any extraneous microparticles to prevent side reactions or additional absorbance. Standards were prepared in 2 M KCl to provide the same matrix.

### ^13^C solid-state nuclear magnetic resonance spectroscopy

Solid-state NMR spectra were collected using a Bruker (Billerica, USA) Avance III 600 MHz spectrometer equipped with a 4.0 mm magic-angle spinning Efree triple resonance HCN probe. 22–29 mg of each sample were packed into 50 µl 4.0 mm zirconia rotors. 1D-^13^C-spectra were acquired using standard Bruker cross-polarization (CP) pulse sequence at 10 or 12 kHz sample rotation frequency and at 27.0 °C VT inlet gas temperature. 24,576 scans were signal averaged for each spectrum with 3 s recycle delay.^13^C polarization was achieved using a ramped-amplitude^1^H-^13^C CP pulse for 2.0 ms, and the spectra were acquired for 15 ms under 81 kHz two-phase-modulated (TPPM)^1^H decoupling.^13^C chemical shifts were referenced externally to 2,2-dimethyl-2-silapentane-5-sulfonate sodium salt using the adamantane methylene peak at 40.48 ppm^40^. All data were processed with applying a 30 Hz exponential line broadening using Bruker Topspin 3.5.

### Gas adsorption

N_2_ and CO_2_ adsorption were combined to characterize the SSA and pore volume (PV) of the biochar samples. To this end, the adsorption and desorption isotherms for both gases were measured at 77 K and 273 K, respectively. Before the measurements, the char samples were pretreated to clean the char surface from other adsorbed species (degassing) by heating them in vacuum at 120 °C during 6 h. The samples were also milled to reduce the influence of transport limitations on the measurements, especially in the case of N_2_ adsorption, due to the low temperatures. The N_2_ isotherms were determined measuring the amount of N_2_ adsorbed/desorbed as function of the N_2_ relative pressure P/P_0_ in the measurement station, where P is the pressure of N_2_ in the measurement station and P_0_ is the N_2_ saturation pressure at the measurement temperature (~77 K). The relative pressure P/P_0_ ranged from ~0.005 to ~0.995. In the case of CO_2_ the relative pressure ranged from ~0.00005 to ~0.028. For both degassing and adsorption/desorption measurements the gas sorption system Nova 2000 provided by Quantachrome Instruments (Boynton Beach, FL, USA) was used. Several methods were applied to the isotherms in order to derive pore surfaces areas and volumes, which are included in the NovaWin software (Quantachrome Instruments). In the case of N_2_ adsorption, the Brunauer, Emmett and Teller (BET)^41^ method was used to determine the total internal SSA of the samples. The DFT (density functional theory)^42^ method was applied to the adsorption isotherm to characterize the pore size distribution, in particular, the QSDFT (quenched solid-state functional theory) method, considering slit/cylindrical pores. With this method, micropores and mesopores up to ~34 nm in pore width could be measured. The Barrett, Joyner and Halenda (BJH)^43^ method was applied to characterize mesopores bigger than ~3 nm and macropores up to the detectable limit of N_2_ adsorption. The total pore volume was determined from the volume of N_2_ adsorbed at a relative pressure (P/P_0_) ≈1, assuming that at this point the pores are filled with liquid N_2_. For CO_2_ adsorption, the nonlinear DFT (NLDFT) was applied to determine the SSA and volume due to micropores.

### X-ray photoelectron spectroscopy

XPS uses X-rays to excite atoms of a sample and analyzes the energy of the emitted photoelectrons, which provides information on both elemental composition and chemical bonds of the outermost 10 nm^44^. XPS was performed on an ESCALAB250Xi (Thermo Scientific, UK) using monochromated Al K α (1486.68 eV, 150 W) at a spot size of 500 µm under high-vacuum conditions (<2×10^−9^ mbar) and a photoelectron takeoff angle of 90° was used. It was calibrated with Au (Au 4f7 = 83.96 eV), Ag (Ag 3d5 = 368.21 eV) and Cu (Cu 2p3 = 932.62 eV). Pass energy was 100 eV for survey scans and 20 eV for region scans. Biochar particles were gently washed with DI water three times, dried at 40 °C and were measured both as a particle and as a powder after grinding in an agate mortar.

### Scanning electron microscopy

In SEM, a focused beam of accelerated electrons scans over the samples and creates a multitude of characteristic secondary emissions. We used secondary electrons, which provide predominantly topographic information of the sample, and EDS. The energy of the X-rays is characteristic for the atom that was excited by a primary electron (beam electron) and thus provides information about the elemental content of the outermost hundreds of nm to ~2 µm of the sample, depending on the composition of the sample and the energy of the incident electron beam^45^. SEM micrographs and X-ray spectra were obtained on a Zeiss Sigma SEM with Bruker EDS at a working distance of 8–9 mm and an acceleration voltage of 10–15 kV. Individual biochar particles were mounted on SEM stubs with conductive carbon paint. Sputter coating was not necessary.

### Scanning transmission X-ray microscopy

Ultra-thin-sections for STXM were prepared by cutting pieces of biochar (no pretreatment, no drying) under cryo-conditions in an ultramicrotome Leica UCT, gluing the sample with a droplet of 2.3 M saccharose and catching the ultra-thin-sections with saccharose, too. They were placed on formvar-coated 200 mesh copper grids. Grids were carefully rinsed with water to remove the saccharose and mounted on a STXM sample plate. Despite the rinsing, saccharose was still present on all samples. Therefore, a reference spectrum of pure saccharose was used to quantitatively map saccharose by spectral decomposition in the samples. Ultra-thin-sections were obtained from both center and the edge of pristine biochar particles as well as the edge of co-composted biochar particles. Measurements were conducted at the Canadian Light Source beamline 10ID-1.

### Analytical scanning transmission electron microscopy

Pieces of biochar were covered in gold all-around in the Leica EM ACE 600 (Leica Microsystems, Vienna, Austria; 45 mA, 8,0 × 10^−3^ mbar, working distance 50 mm) using a rotating device to enable 3D coating. This resulted in a minimum of 25-nm-thick gold layer on the biochar. These nuggets were embedded in Spezifix40 (Struers, Willich, Germany) and after hardening carefully trimmed with a trim 90 blade (Diatome, Switzerland). The preparation of 50 nm slices was done at room temperature with the ultra-sonic-knife (Ultra Sonic, Diatome) for reducing compression and allowing best structure preservation in the ultramicrotome Leica EM UC6 (Leica Microsystems). The slices were transferred from the water with a perfect loop (Diatome) on a 200 mesh grid. STEM investigations were performed on a monochromated probe corrected FEI Titan G2 60–300 (STEM) microscope with an X-FEG Schottky field-emission electron source operated at 60 kV. The microscope is equipped with a FEI Super-X detector (Chemi-STEM technology), consisting of four separate silicon drift detectors^46^ (120 mm acquisition area) and a Dual EELS – Gatan Imaging Filter (GIF) Quantum^47^. The microscope was run in monochromated mode with a spatial resolution of 0.1 nm and an energy resolution at full-width at half-maximum of 0.17 eV. Two different detectors: high angular annular dark field (HAADF) and annular dark field (ADF), a beam current of approximate 120 pA and acquisition times ranging from 20 to 30 μs/pixel were used for micrographs acquisition. Analytical investigations involved electron energy loss (EELS) and X-ray (EDX) spectroscopy in STEM mode by line scans and spectrum imaging, which, however, were acquired with different acquisition times and pixel sizes depending on the investigated area. Energy loss near edge structure (ELNES) of carbon-, nitrogen- and oxygen- K ionization edges were analyzed considering the specific finger prints and energy shifts of different types of bonding which might be present in the sample, with graphite as a reference. Elemental quantification has been performed by using the k-factor method^45, 48^. Sample thickness in different areas was determined by EELS^48^. However, for the mean free path of the sample with the major constituent carbon a calculated value of 76.6 nm (Malis formula^48^) was used. The images and spectra were recorded by a Gatan Digiscan unit and Digital Micrograph software and were corrected for dark current and gain variations.

### Desorption atmospheric pressure photoionization FT-ICR MS

A modified ThermoFisher LCQ atmospheric pressure photoionization (APPI) source (ThermoFisher Corp., Bremen, Germany) was used for all Fourier-transformed ion cyclotron resonance mass spectrometry (FT-ICR MS) experiments^49^. Pieces of pristine and co-composted biochar were held by tweezers ∼ 1 mm from the exit of the heated ceramic nebulization tube and ~10 mm from the FT-ICR MS inlet. Gas-phase neutrals were produced through a combination of thermal and chemical desorption^50^, and ionized by dopant-assisted APPI^51^. Nitrogen was used as nebulizer gas at 100 psi with toluene as dopant at a flow rate of 50 μl min^−1^. The temperature of the heated nebulizer gas/solvent plume ranged from 100 to 500 °C depending upon the sample. Mass spectra were acquired by a custom-built FT-ICR MS based on a passively shielded 9.4 T horizontal 200 mm bore diameter superconducting solenoid magnet (Oxford Corp., Oxford Mead, UK) operated at room temperature^52^. A modular ICR data station (Predator) facilitated instrument control, data acquisition, and data analysis^53^. Positive ions were accumulated in an external quadrupole for 50–500 ms, and passed through an rf-only quadrupole into an octopole equipped with tilted wire extraction electrodes for improved ion extraction and transmission^54^. Helium gas was introduced into the octopole to collisionally cool the ions before transfer through rf-only quadrupoles (total length 127 cm) into a seven-segment open cylindrical ICR cell with capacitively-coupled excitation electrodes based on the Tolmachev configuration^55^. Approximately 25–50 time-domain acquisitions were co-added, Hanning-apodized, and zero-filled once prior to fast Fourier transform and magnitude calculation. ICR frequencies were converted to *m/z* values by the quadrupolar electric trapping potential approximation^56^. Spectra were internally calibrated from abundant homologous alkylation series (compounds that differ in elemental composition by integer multiples of CH_2_). Mass spectral peaks with signal magnitude greater than six times the baseline root-mean-square (r.m.s.) noise level were assigned elemental composition values (C_0–100_H_0–200_N_0–5_O_0–15_S_0–2_) with PetroOrg software (Y.E. Corilo; Florida State University; all rights reserved), subject to the following constraints for chemical feasibility: mass error < 1 p.p.m.; the computed ring and double bond equivalent must be an integer value ≥ 0; neutral formulas containing an odd number of nitrogen atoms must have an odd nominal mass, and neutral formulas containing zero or an even number of nitrogen atoms must have an even nominal mass (nitrogen rule); 2 ≤ H ≤ (2 C + 2); 0.33 ≤ H/C ≤2.25; 0 ≤ O ≤ (C + 2); O/C <1.2; N/C <0.5; S/C <0.2^57^.

DAPPI FT-ICR MS was performed both on original biochar samples and on washed biochar as described below. However, data on the washed samples was more comprehensive and better calibrated, i.e., more detected masses could be assigned to formulas. In the original co-composted biochar, 6,063 detected masses were not assigned formulas, while only 4,350 could not be assigned after washing.

### Biochar washing for liquid extraction of the organic coating

Biochar particles were washed in four steps to remove any compost induced surface alterations and, at the same time, to obtain a suspension of the co-composting derived material that forms the coating of the biochar as identified with STEM. 15 mL DI water were added to 2.5 g moist biochar in a Falcon tube (step 1). After 5 min on a roller shaker at 50 rpm (Roller 10 digital, IKA, Staufen, Germany), water was decanted through a 0.5 mm mesh sieve and 15 ml of DI water were added to repeat this procedure (step 2). After decanting, 15 ml of 0.05 M NaOH solution were added as suggested by Tsechansky and Graber^28^ and Falcon tubes were placed on the shaker for 90 min (step 3). After decanting, 15 ml of DI water were added for 20 min to remove residual NaOH (step 4). Separate washing procedures were conducted for fresh and co-composted biochar. Afterwards, the biochar was dried at 40 °C for 72 h for DAPPI FT-ICR MS, SEM and STEM as described above. After washing, co-composted biochar was almost black again (Supplementary Fig. 7). Washed biochar was analyzed using SEM and STEM to verify the removal of the coating. SEM showed that the original biochar surface structure was widely exposed again, while few regions still showed surface alterations (Supplementary Fig. 8). In STEM we could not identify a coating after washing, but granular aggregations of Ca and O were present on the biochar surface (Supplementary Fig. 9).

Washing solution of step 3 (0.05 M NaOH) was analyzed by FTIR,^1^H NMR, excitation–emission matrix (EEM) fluorescence spectroscopy, ion chromatography, and for dissolved organic and inorganic carbon (DOC/DIC) and electron accepting and donating capacity (EAC/EDC). Before analysis, resulting washing solutions were filtered through a 0.45 µm syringe filter. For FTIR, washing solutions and original 0.05 M NaOH solution were freeze-dried. For^1^H NMR, washing procedure was repeated and 0.05 M NaOH was prepared with D_2_O instead of H_2_O. Major anions and cations were quantified by ion chromatography (IC, DX-120, Dionex, Sunnyvale, CA, USA). DOC + DIC was quantified on a Vario cube (Elementar, Hanau, Germany), DOC was quantified on the same device after acidifying the samples to pH 1 with HCl. DIC was calculated as [DOC + DIC]—[DOC].

### Excitation–emission matrix

EEM fluorescence spectra of washing solution were analyzed using a Fluoromax4 (Horiba, Jobin-Yvon) spectrofluorometer. EEM fluorescence spectra were recorded over a range of excitation wavelength (300–500 nm) and emission wavelength (400–600 nm) relevant for natural organic matter^58^.

### Electron accepting and donating capacity

The redox properties, i.e., electron accepting and donating capacities (EAC/EDC) of biochar washing solutions were determined by mediated electrochemical reduction (MER) and oxidation (MEO) following established procedures^6, 59^. In brief, the electrochemical system consists of a glassy carbon cell (Sigradur G, HTW, Germany) as working electrode, a Ag/AgCl as reference electrode (Bioanalytical systems Inc., USA) and a platinum wire (0.5 mm, 99.9 %, Sigma-Aldrich Co., USA) attached to a platinum gauze (52 mesh, 99.9 %, Sigma-Aldrich Co., USA) as auxiliary electrode. The applied potential was measured against Ag/AgCl electrode but is reported against standard hydrogen electrode (EH = −0.49 V in MER and EH =  + 0.61 V in MEO). Electron transfer mediators were used during the measurements to ensure a fast electron transfer between the electrodes and the analyte^59^; diquat-dibromide monohydrate (DQ, Sigma-Aldrich Co., USA) was used for MER and 2,2′-azino-bis(3-ethylbenzothiazoline-6-sulfonic acid) diammonium salt (ABTS, Sigma-Aldrich Co., USA) for MEO. EAC and EDC are calculated from the measured reductive and oxidative currents considering normalization to carbon content (µmol e^-^/mmol C)^6^. The total EEC is the sum of the individual accepting and donating capacities (EEC = EAC + EDC). As an additional reference, dissolved organic matter was extracted from non-biochar-amended control compost in analogy to the biochar washing procedure (0.05 M NaOH, 1.5 h, same shaker).

### Fourier-transformed infrared spectroscopy

Fourier-transformed infrared (FTIR) Spectroscopy provides information on bonding modes in organic molecules by absorption of infrared radiation, which depends the vibrational response of the functional groups^60^. FTIR absorbance spectra of KBr pellets prepared with 0.2% biochar, 0.4% freeze-dried washing solutions or 0.4% freeze-dried control compost were measured with a Vertex 80 v (Bruker) with 128 scans. A KBr pellet without sample was used for background measurements.

### ^1^H solution nuclear magnetic resonance spectroscopy

2 ml of the pristine and the co-composted biochar washing solutions were lyophilized (freeze-dried) overnight. The dry samples were rehydrated up to 500 µl using 100% D_2_O. NMR spectra were collected using a Bruker Avance III 600 MHz spectrometer equipped with a Bruker 5 mm SmartProbe^TM^.^1^H 1D spectra were acquired for 2 s with a 20 s recycle delay and 1024 scans using standard Bruker pulse sequence with water suppression. All spectra were processed with applying a 0.30 Hz exponential line broadening using Bruker Topspin 3.5.

### Fast field cycling nuclear magnetic resonance relaxometry

1 g of each biochar sample was suspended in 3 g of MilliQ grade water (resistivity of 18.2 MΩ cm at 298 K). Milli-Q water was produced by a Milli-Q Advantage A10 Ultrapure Water Purification System (Millipore Corporation, Massachusetts, USA). The suspensions were allowed to sediment overnight prior to the relaxometry investigations. The samples were put in the probe of a Stelar SpinMaster FFC-2000 fast field cycling relaxometer (Stelar s.r.l., Mede, PV−Italy) and analyzed at 25 °C. The basic theory about FFC NMR relaxometry and the sequence applied for the experiments reported in this study have been already summarized by Conte and Alonzo^31^. In brief, non-polarized (NP) and polarized (PP) sequences were applied. In the NP sequence, a relaxation field (*B*_RLX_) was changed between proton Larmor frequency (*ω*_L_) values ranging in the interval 0.01–30 MHz. Each *B*_RLX_ was applied for a period *τ* arrayed with 32 logarithmic spaced time sets, each of them adjusted at every relaxation field in order to optimize the sampling of the decay/recovery curves. At the end of each *B*_RLX_ field a^1^H 90° pulse was applied into an acquisition magnetic field (*B*_ACQ_) held for a fixed time at the *ω*_L_ value of 7.2 MHz. The ^1^H 90° pulse was needed to make magnetization observable and the free induction decay acquirable. A time domain of 100 µs sampled with 1000 points was applied. Field-switching time was 3 ms, while spectrometer dead time was 15 µs. For all of the experiments a recycle delay of 20 s was used. In the PP sequence, a polarization field (*B*_POL_) set at the *ω*_L_ of 9 MHz was applied prior to each *B*_RLX_ field. The period of time during which *B*_POL_ was applied (referred to as polarization time or *T*_POL_) corresponded to five times the *T*_1_ estimated at this frequency.

The crossover field between NP and PP sequences was approximately retrieved when the relaxation field intensity was half of that of the polarization field^31^.

### FFC NMR relaxometry data elaboration

The longitudinal relaxation time (*T*_1_) values of the observed nuclei were obtained for each *B*_RLX_ by changing the *τ* values as reported above. The relationship between signal intensity and *τ* is modeled by Eq. 1:

1 $$I\left( \tau \right) = {I_0}\exp {\left[ { - \left( {\tau {\rm{/}}{T_1}} \right)} \right]^k}.$$

Here, *I*(*τ*) is the ^1^H signal intensity at each fixed *B*_RLX_, I_0_ is the ^1^H signal intensity at the thermal equilibrium, *T*_1_ is the average proton spin-lattice relaxation time and *k* is a heterogeneity parameter related to the stretching of the decay process. This function, which accounts for the large sample heterogeneity resulting in a multi-exponential behavior of the decay/recovery curves^31^, can be considered as a superposition of exponential contributions, thereby describing the likely physical picture of some distribution in *T*_1_. Eq. 1 has the advantage that it is able to handle a wide range of behaviors within a single model. For this reason, assumptions about the number of exponentials to be used in modeling nuclear magnetic dispersion (NMRD) data are not necessary. The NMRD profiles resulting from the elaboration of the decay/recovery curves are reported in Supplementary Fig. 13. The data acquired at the relaxation field of 0.01 MHz were transformed by applying the Uniform PENalty regularization (UPEN) algorithm^31^ which allowed the achievement of the distribution of the longitudinal relaxation times (also referred to as relaxograms).

### Liquid chromatography organic carbon detection

1 g of pristine, co-composted and soil-aged biochar was added to 10 ml of distilled water at 50 °C for 24 h and regularly stirred, and then centrifuged and filtered (10,170 *g*, 5 min, 0.45 µm) to separate the solid and liquid phases. The supernatant solutions were analyzed by Liquid Chromatography – Organic Carbon Detection LC-OCD model 8 (DOC Labor, Germany). Details of the measurement procedure have been described in full by Huber et al^61^. In this study, a Toyopearl TSK HW50S LC column was used with a phosphate buffered mobile phase of pH 6.4 at a flow rate of 1.1 ml min^-1^. Injection volumes were 1,000 µl. The supernatant solutions are also analyzed to determine total organic nitrogen and carbon and total inorganic carbon using a thermocatalytic high-temperature oxidation process (Multi N/C 2100, Analytic Jena, Germany).

The chromatographic eluent subdivides into six sub-fractions, biopolymers, humics, building blocks, low molecular-weight neutrals and hydrophobic organic carbon (Supplementary Table 5), as suggested by Huber et al.^61^

### Data availability

The data sets generated during and/or analyzed during the current study are available from the corresponding author on reasonable request.

## Electronic supplementary material

Supplementary Information

Peer Review File
