# Supplementary material for: Organic coating on biochar explains its nutrient retention and stimulation of soil fertility
Source: Nat Commun. 2017 Oct 20;8:1089. doi: 10.1038/s41467-017-01123-0 (PMC5715018; doi:10.1038/s41467-017-01123-0)
Supplement: Supplementary file 1 — Supplementary Information [file 41467_2017_1123_MOESM1_ESM.pdf]

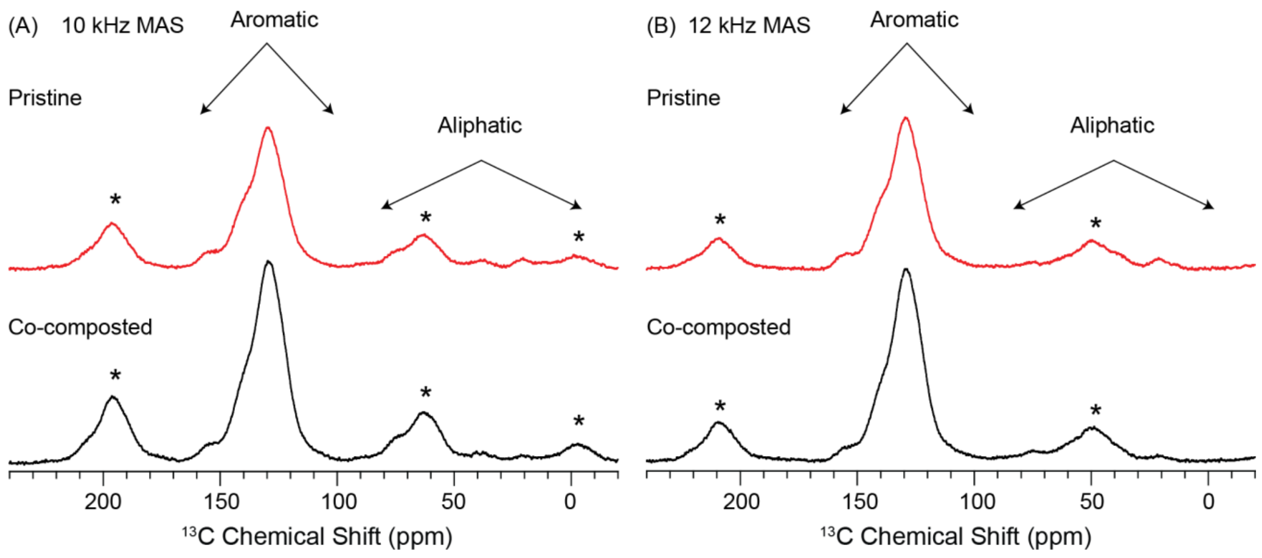

Supplementary Fig. 1: ^{13}C solid-state Nuclear Magnetic Resonance (NMR) spectra of biochar. ^{13}C solid-state NMR spectra of pristine and co-composted biochar were acquired to determine whether there were any significant differences in the composition of the two samples on the bulk level. Pristine (top, red) and co-composted (bottom, black) biochar were analyzed at 10 kHz (A) and 12 kHz (B) magic angle spinning (MAS) frequency to distinguish potential weak signals in the aliphatic region from the spinning sidebands. The spinning sidebands (which occur at multiples of the spinning frequency) are denoted with asterisks. The dominant, broad peak in the 100-165 ppm region corresponds to aromatic or unsaturated compounds in both samples. Low intensity aliphatic peaks are also observed in the 10-60 ppm region. The spectra have poor resolution, however they did not indicate any major differences between the pristine and co-composted samples.

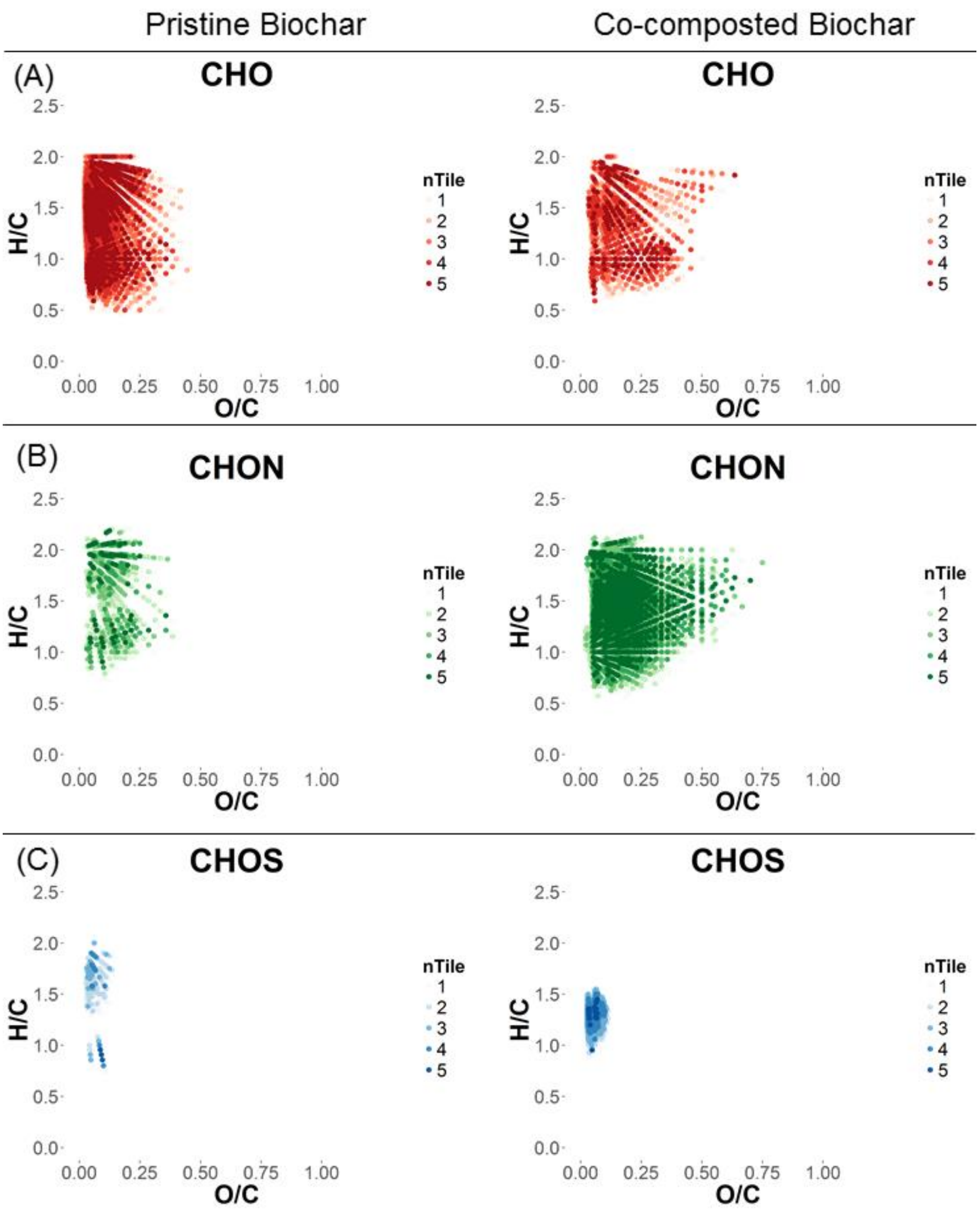

Supplementary Fig. 2: Pristine and co-composted biochar analyzed with Desorption Atmospheric Pressure Photoionization Fourier Transform Ion Cyclotron Resonance Mass Spectrometry (DAPPI FT ICR MS). Van Krevelen diagrams of compounds assigned to the (A) CHO class ($C_xH_yO_z$), (B) CHON ($C_WH_XO_yN_z$) class, and (C) CHOS class ($C_WH_XO_yS_z$). Each dot represents 1-5 compounds measured, depending on depth of color according to the legend.

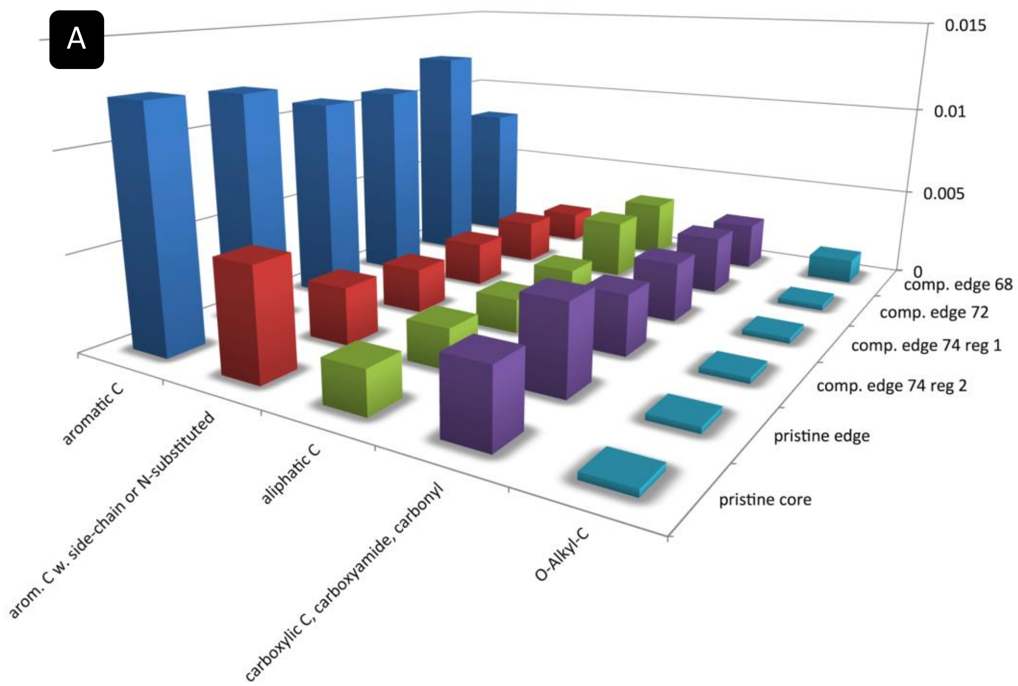
A
0.015
0.01
0.005
0
comp. edge 68
comp. edge 72
comp. edge 74 reg 1
comp. edge 74 reg 2
pristine edge
pristine core
aromatic C
arom. C w. side-chain or N-substituted
aliphatic C
carboxylic C, carboxyamide, carbonyl
O-Alkyl-C

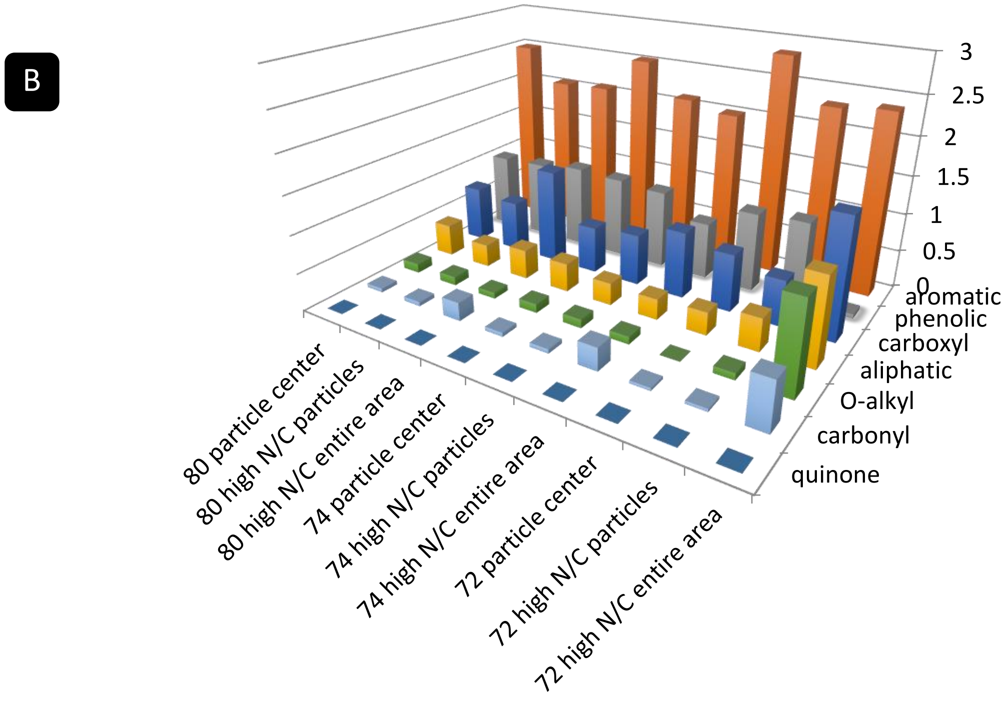
B
3
2.5
2
1.5
1
0.5
0
aromatic
phenolic
carboxyl
aliphatic
O-alkyl
carbonyl
quinone
80 particle center
80 high N/C particles
80 high N/C entire area
74 particle center
74 high N/C particles
74 high N/C entire area
72 particle center
72 high N/C particles
72 high N/C entire area

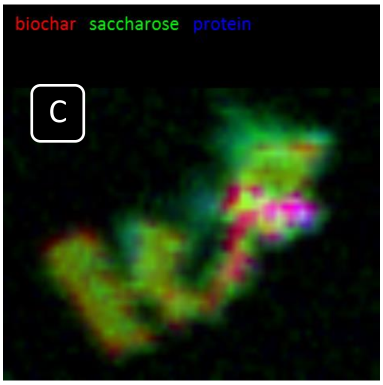
biochar saccharose protein
C

Supplementary Fig. 3: Co-composted biochar analyzed with Scanning Transmission X-ray Microscopy (STXM). (A) Normalized contributions of different functional groups to the X-ray absorption across the C1s absorption edge. These semi-quantitative results are based on spectral decomposition of X-ray absorption spectra that were each normalized to a 1 nm layer and cobtained from different ultrathin-sections of biochar particles. “Pristine core” refers to an ultrathin-section cut from the center of a pristine biochar particle after removing the outside by trimming. “Pristine edge” refers to an ultrathin-section cut from a pristine biochar particle as close to the outer surface as possible. “Comp edge” refers to an ultrathin-section obtained from a co-composted biochar particle as close to the outer surface as possible.

(B) Normalized relative contributions of different functional groups to the X-ray absorption across the C1s absorption edge in ROIs defined according to their N/C ratio as described in Figure 4. These semi-quantitative results are based on spectral decomposition of X-ray absorption spectra that were each normalized to a 1 nm layer and obtained from three regions of interest in different spots; spot 74 is shown in Figure 4 of the main manuscript. All high N/C ROIs showed a lower relative abundance of aromatic. No consistent trends could be described for aliphatic and o-alkyl carbon. In high N/C entire area ROIs, relatively more carboxyl C was found as well as at least 4 times higher abundance of carbonyl C and less phenol C than in particle ROIs. Redox-active quinone C hardly showed a consistent trend, but in one region (72), we found a 6 times higher abundance of quinone C in the high N/C particle ROI. In the spectral decomposition, no consistent trend was found that would indicate a significant presence of carbonates, that would result in an absorption peak at 290.3 eV through the 1s → σ* excitation.

(C) RGB-map showing the distribution of biochar, saccharose and protein, calculated from an image stack across the C1s absorption edge through linear spectral decomposition with the spectra of three reference compounds. Saccharose is a contamination as a result of sample preparation. Protein was used as an exemplary N-rich carbonaceous compound. Scale bar represents 500 nm.

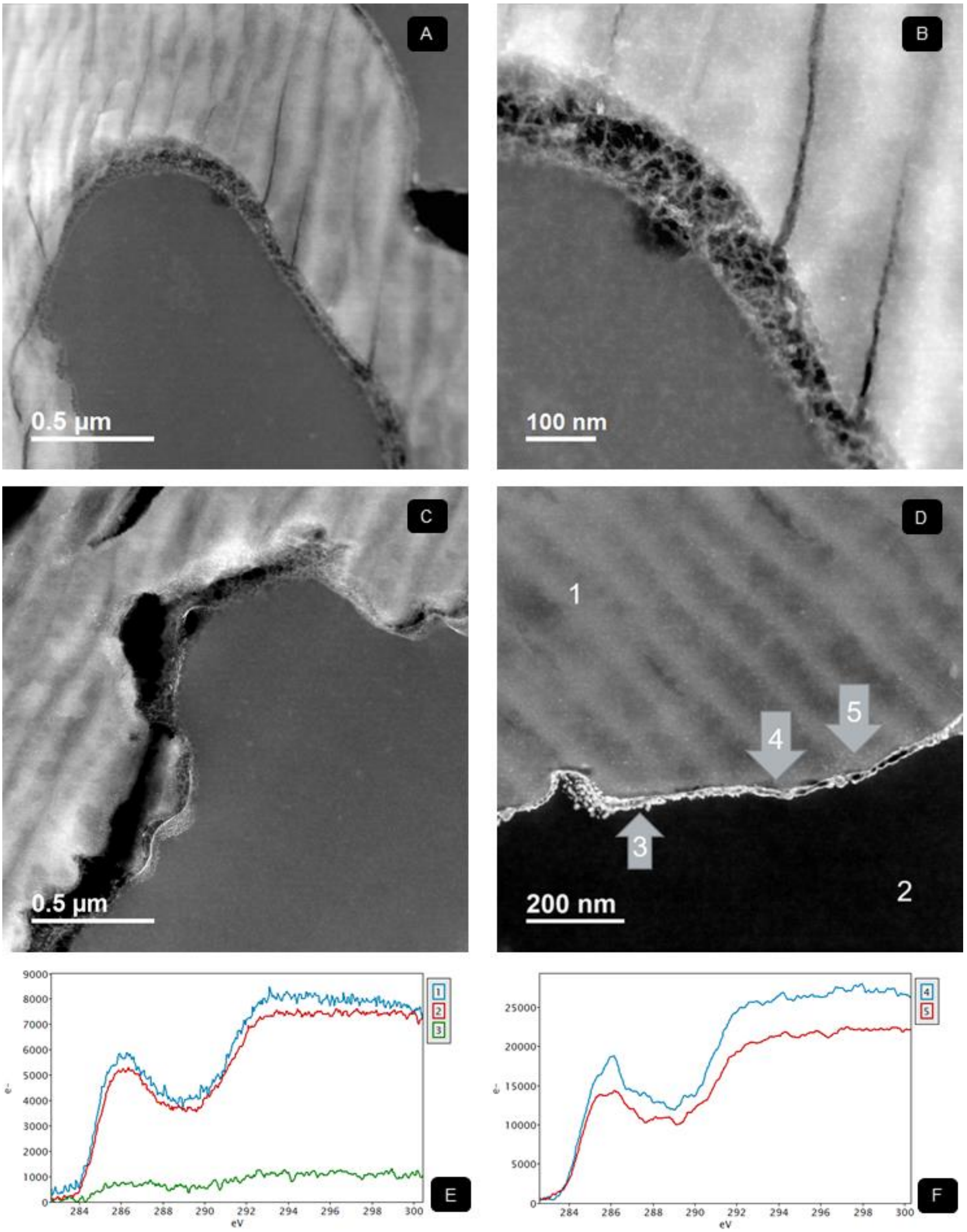

Supplementary Fig. 4: STEM HAADF (Scanning transmission electron microscopy – high angle annular dark field) micrographs and EELS (electron energy loss spectroscopy) spectra of ultra-thin sections of co-composted biochar.

(A): Thick organic coating on surface that are most likely located inside a biochar pore as indicated by only thin gold coating that is visible as individual bright spots. Rippled appearance of the biochar is an artefact of the ultrasonic knife used in the ultramicrotom. As the biochar is more brittle than the glue and the coating, it is only the biochar to show this artefact.

(B) Close-up of (a) on a thick (100 nm) and intact part of the coating highlighting its porous nature.

(C): Overview on a partly delaminating coating; delamination is an artefact of the mechanical force applied during microtoming. It shows the three-dimensional nature of the porous coating and that delaminating preferentially separates biochar and coating.

(D) Thin organic coating on outer surface of co-composted biochar: (1) Biochar with artefact from oscillating diamond knife; (2) Epoxy resin; (3) Sputter-coated gold layer (bright dots and layers); (4) Dark area between gold and biochar, organo-mineral coating, here > ~ 10 nm, rich in O and N as shown by EELS Spectra (data not shown). (5) Location of bright dots, identified by EELS as hotspots of mineral matter, predominantly Ca (data not shown).

(E, F): EELS spectra obtained at respective locations marked in (D):

> (E): Spectrum of the epoxy resin is similar to the spectrum of the biochar matrix, which highlights the need for the gold coating to clearly differentiate biochar and resin.
>
> (F): Spectrum of the N-rich coating (4) shows an alteration of the C-K near edge fine structure in the C=C 1s–π^* transition of aromatic carbon. Spectrum in the area of the bright Ca hotspots (5) hardly shows alteration compared to the biochar spectrum (e)/(1).

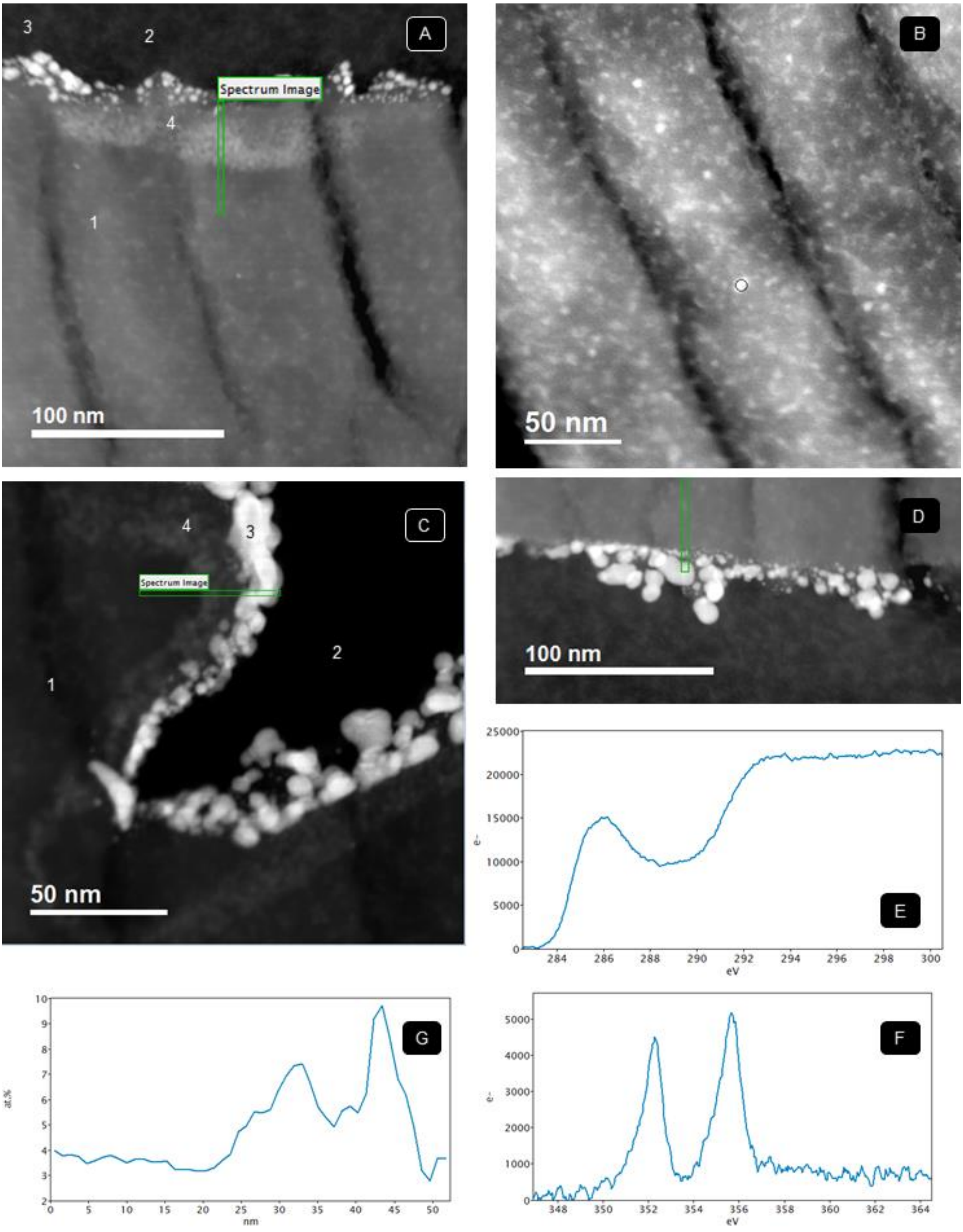

Supplementary Fig. 5: STEM HAADF (Scanning transmission electron microscopy – high angle annular dark field) micrographs and EELS (electron energy loss spectroscopy) spectra of ultra-thin sections of pristine biochar.

(a) STEM HAADF micrographs of an ultra-thin section of pristine biochar. Rippled appearance of the biochar (1) is an artefact of the ultrasonic knife used in the ultramictom. As the biochar is more brittle than the epoxy resin (2), it is only the biochar to show this artefact. High abundance of gold (3) that is not high enough to form a continuous coating indicates a semi-exposed location of this spot in the original biochar particle. No porous coating could be detected. (4) indicates a region of bright spots that show a high content of Ca according to EDX (data not shown).

(b) Close-up on the biochar matrix show the rippled appearance and the ubiquitous presence of bright dots in the nm range. EDS revealed their higher content of inorganic matter compared to the bulk biochar matrix, including Si, Ca, P, As, Cu. As is an omnipresent trace-metal, that can be present in low concentrations in all kind of biomass.

(c) Close-up on a region of biochar (1) with up to 15 nm of continuous gold coating (3) indicating a more exposed location of this region in the original biochar particle ((2) = resin), which contains a region with high abundance of Ca-nano-hotspots as described in (a). No porous coating detectable.

(d) Surface region of biochar without detectable porous coating and without Ca hotspots.

(e) Summarized C-K near edge EEL spectrum obtained in a line scan located in (c).

(e) Summarized Ca-L2, 3 near edge EEL spectrum obtained in a line scan located in (c).

(g) Spatial distribution of Ca concentrations in the biochar matrix along the line scan located in (c) revealing that the bright spots contain up to 9-10% of Ca atoms according to EELS.

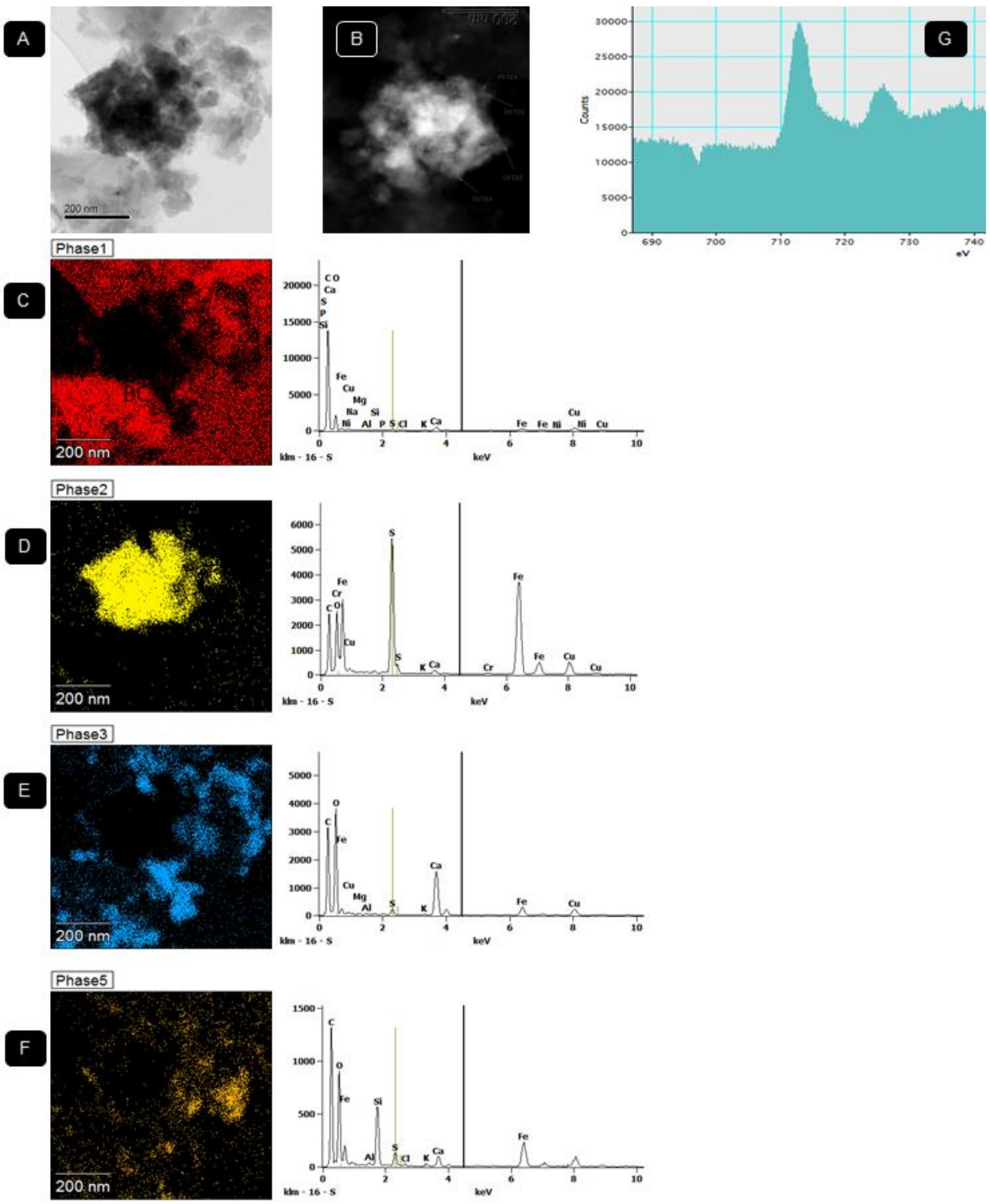

Supplementary Fig. 6: STEM (Scanning transmission electron microscopy) micrographs and EDS (energy dispersive X-ray spectroscopy) maps of co-composted biochar after grounding.

(A) STEM bright field micrograph of an organo-mineral complex.

(B) STEM HAADF (high angle annular dark field) micrograph of the area that illustrates the high degree of porosity of the organo-mineral complex.

(C-F) Phase maps and respective EDS summary spectrum of the highlighted region/phase.

(C) Organic phase, rich in C, with traces of various elements.

(D) Phase dominated by Fe/S/O.

(E) Phase dominated by Ca/O.

(F) Phase dominated by Si/O.

(G) The EELS spectrum of the Fe/O hotspots in (a/b) indicating the presence of magnetite, a mixed-valent iron oxide.

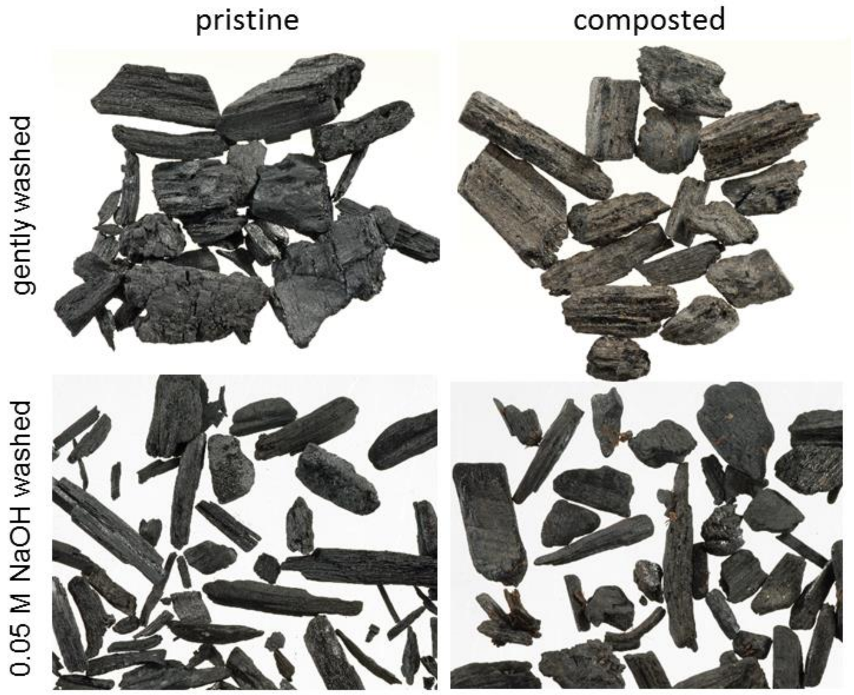

Supplementary Fig. 7: Photographs of pristine and co-composted biochar before and after washing with 0.05 M NaOH. All samples were gently washed with DI water for 10 min to remove loosely attached compost particles. Note the brownish color of the co-composted biochar (top right), while co-composted biochar after washing was almost completely black again.

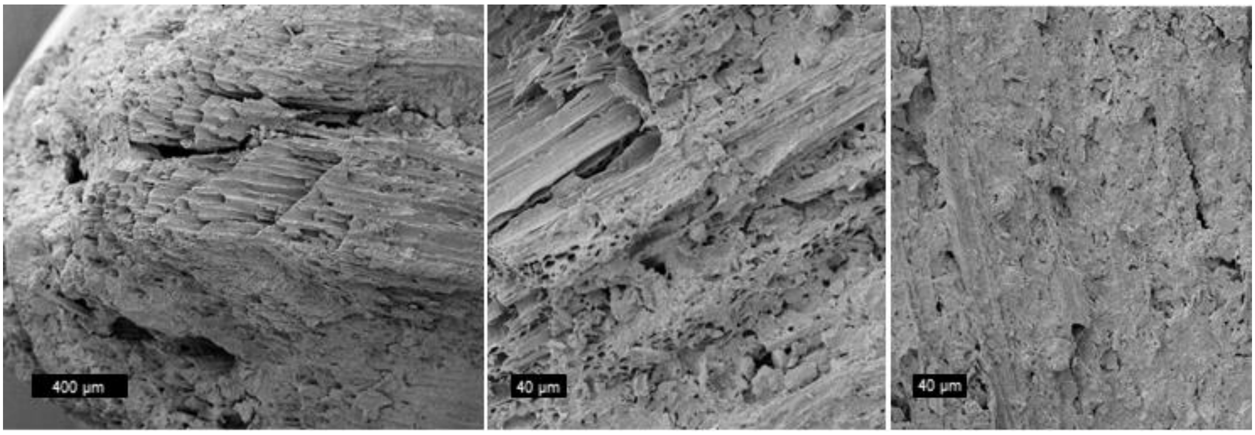

Supplementary Fig. 8: SEM (Scanning electron microscopy) micrographs of co-composted biochar after washing. Original structure of the biochar is visible after washing, there are no thick and large scale coatings detectable. However, some surface alterations are still detectable that cover the original biochar structure (right micrograph).

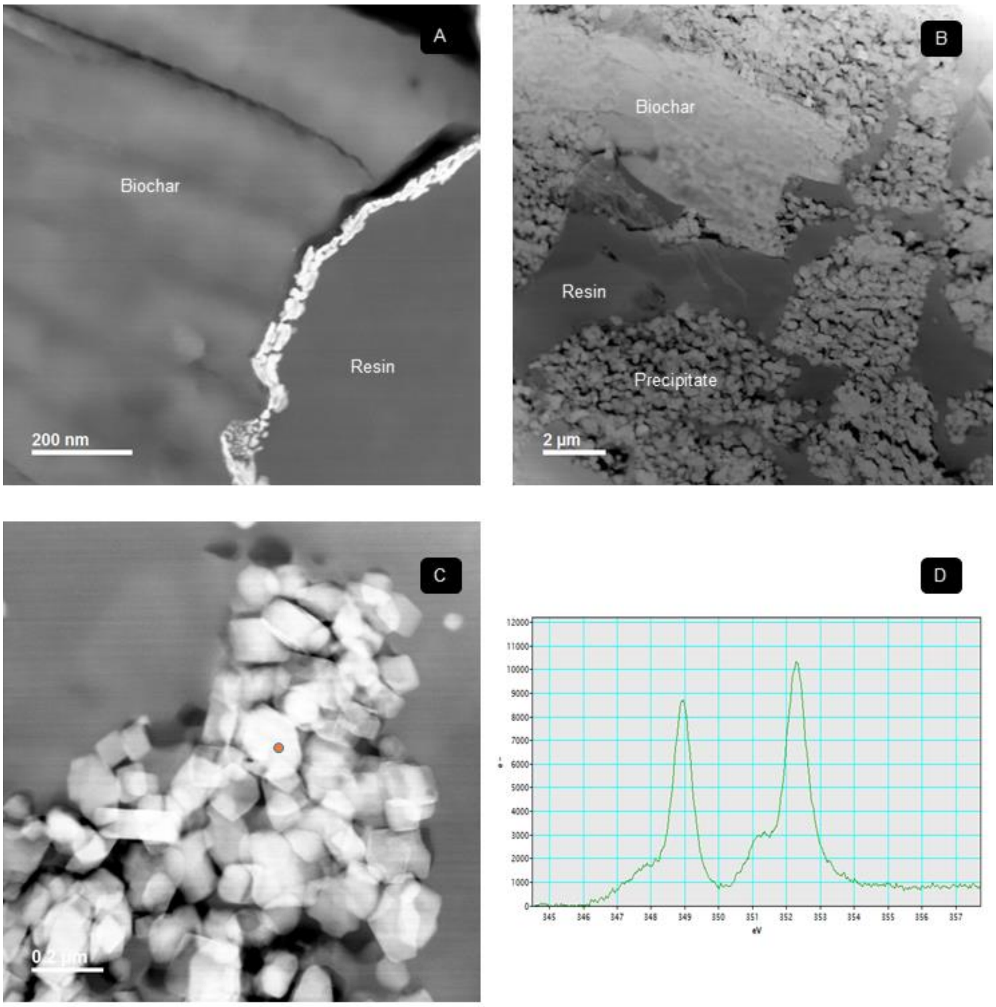

Supplementary Fig. 9: STEM HAADF (Scanning transmission electron microscopy – high angle annular dark field) micrographs and EELS (electron energy loss spectroscopy) spectra of ultra-thin sections of co-composted biochar after 1.5 h washing in 0.05 M NaOH. (A) Biochar surface without organo-mineral coating under a thick layer of gold. Biochar broke within its matrix and right under the gold coating as an artefact of preparation. (B) Overview on an ultra-thin section showing the abundant granular material that is partially associated with biochar surfaces. (C) Close-up on the granular material. (D) EELS spectrum of the Ca-L2,3 region taken at the position marked in (D). We could not identify a porous coating but granular aggregations of Ca and O. Neither the washing nor the preparation of the samples did not involve calcium-containing solutions. We speculate that the 0.05 M NaOH solution removed (dissolved, suspended) the organo-mineral coating, but also mobilized calcium from the biochar. The biochar used in this study contained 3.7% Ca (Supplementary Table 1) that was shown to be concentrated near the biochar particle surface (Fig 5E). Ca potentially precipitated as the pH dropped when the washing eluate (0.05 M NaOH, pH 10) was replaced with de-ionized water as a fourth and final washing step.

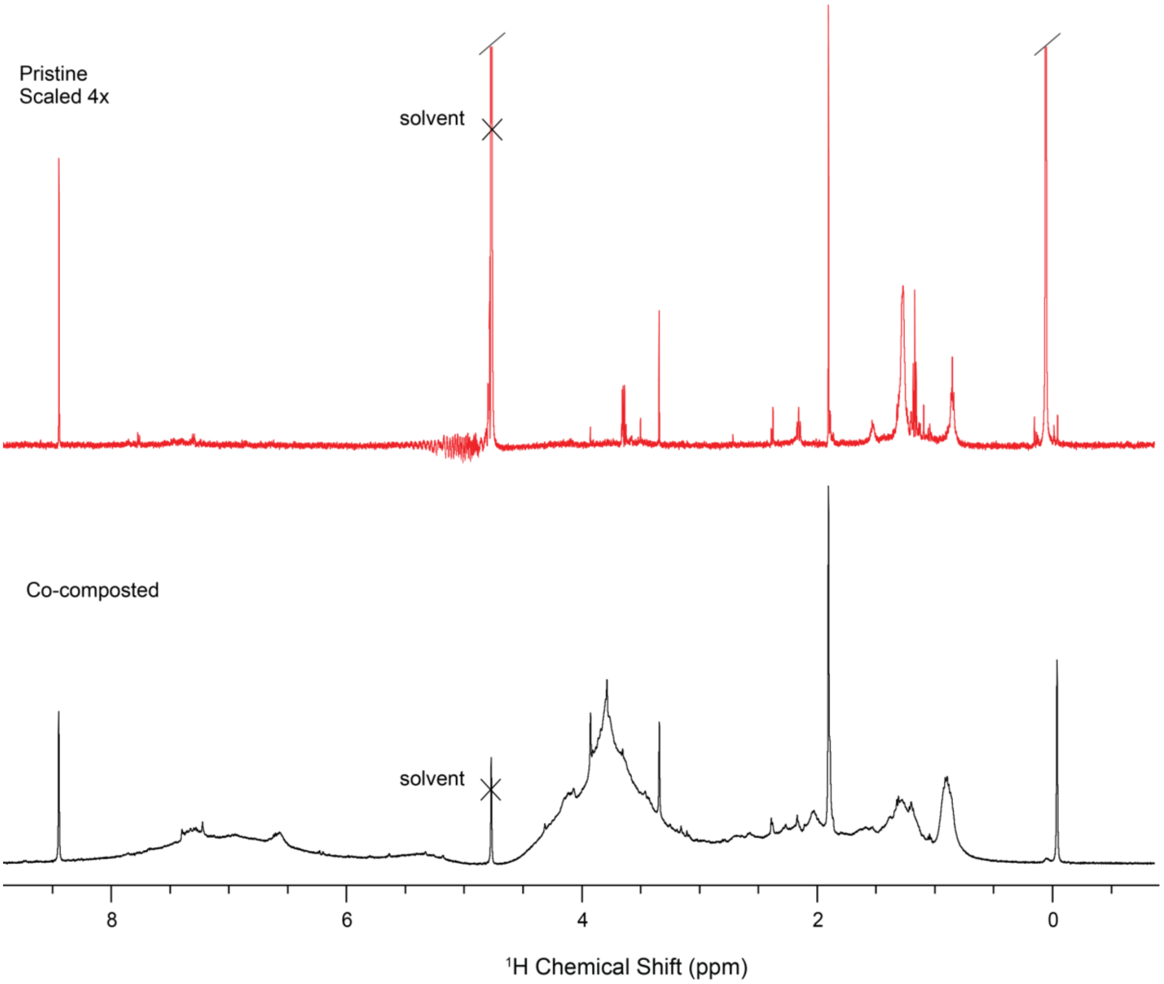

Supplementary Fig. 10: ^{1}H NMR spectra of biochar washing solution. ^{1}H solution NMR spectra of pristine (top, red) and co-composted (bottom, black) biochar washing solution were acquired. The spectrum of the pristine biochar washing solution was scaled 4-fold vertically, and two peaks were truncated. The spectrum of the pristine biochar washing solutions is relatively simple with a few peaks implying the presence of only trace amount of ^{1}H containing organic material. The spectrum of the co-composted biochar washing solution indicates a complex mixture, as the majority of ^{1}H signal appear as broad overlapping peaks. Signals below 1.5 ppm belong to saturated hydrocarbons residues (CH, CH_2, CH_3) that are not attached either to any electron withdrawing group. ^{1}H NMR signals between 1.8 and 2.6 ppm correspond to CH_x structural motifs attached to a carbon of an electron withdrawing groups (*i.e.* protons attached to Cα of ketones, esters, etc.) or to an aromatic ring. Signals between 3.2-4.5 ppm correspond to CH_x structural motifs attached to heteroatoms of electron withdrawing groups (e.g. CH_x-O) implying the presence of acyclic and cyclic ethers, esters or alcohols. There are small amounts of H substituted aromatics and deshielded olefins (δ = 6.5-7.9 ppm).

A)

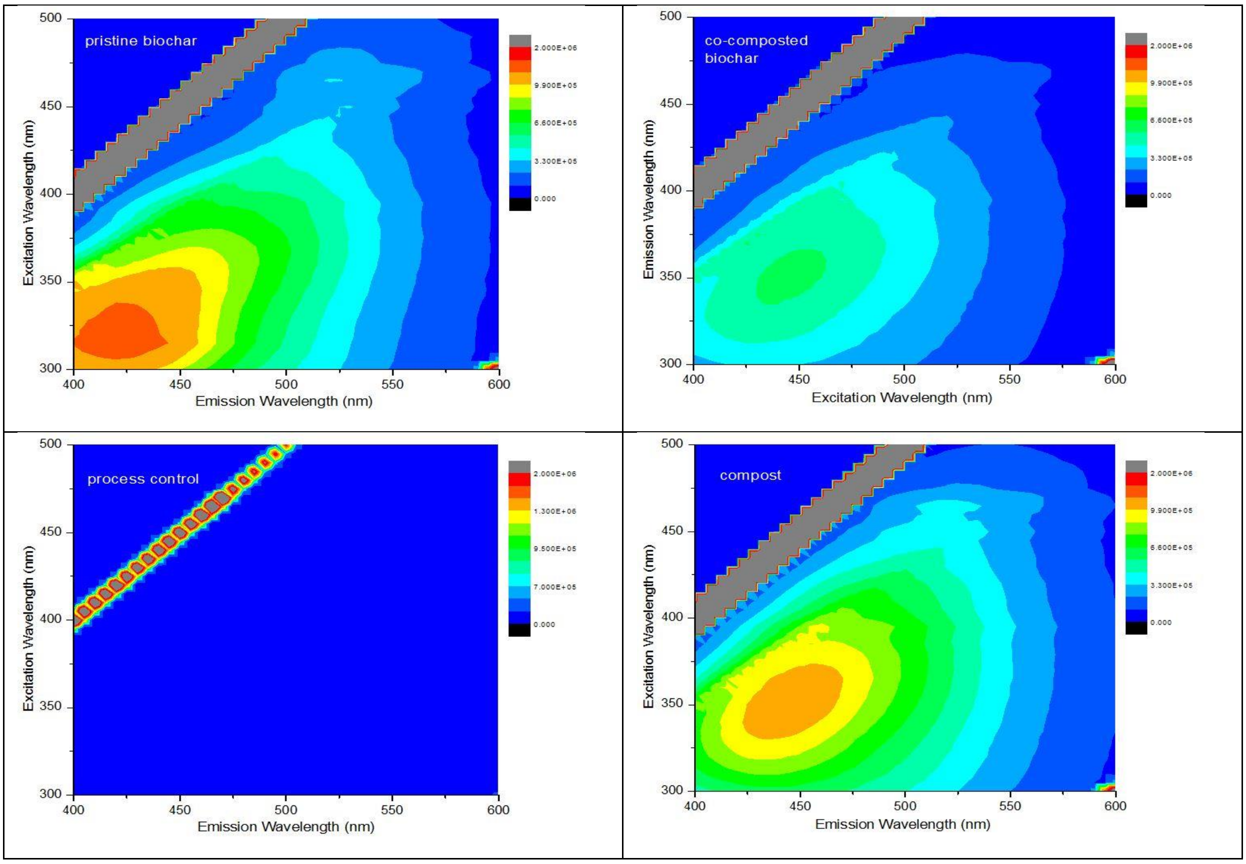

B)

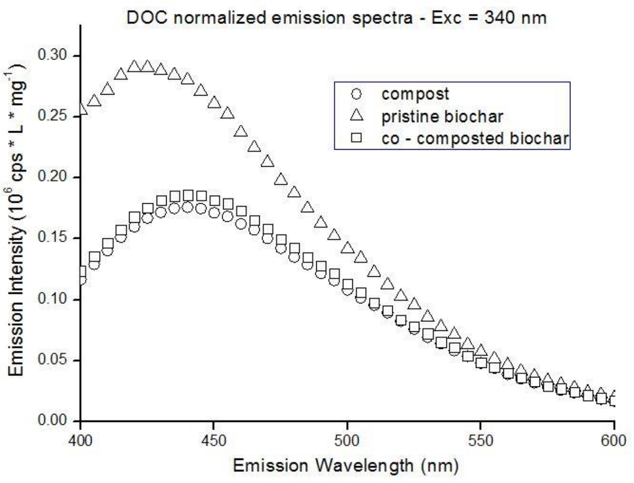

Supplementary Fig. 11: Spectrofluorescence of biochar washing solution: (A) EEM fluorescence maps (excitation wavelength: 300-500 nm; emission wavelength: 400-600 nm). (B) Fluorescence emission spectra at fixed excitation at 340 nm normalized to DOC content. Emission maximum of pristine biochar was shifted from ~425 nm (emission) for pristine biochar to ~ 435 nm (emission) for co-composted biochar, which coincides with the emission maximum of the compost extract and also, showing an incidence of the compost on the emission intensity of the co-composted biochar.

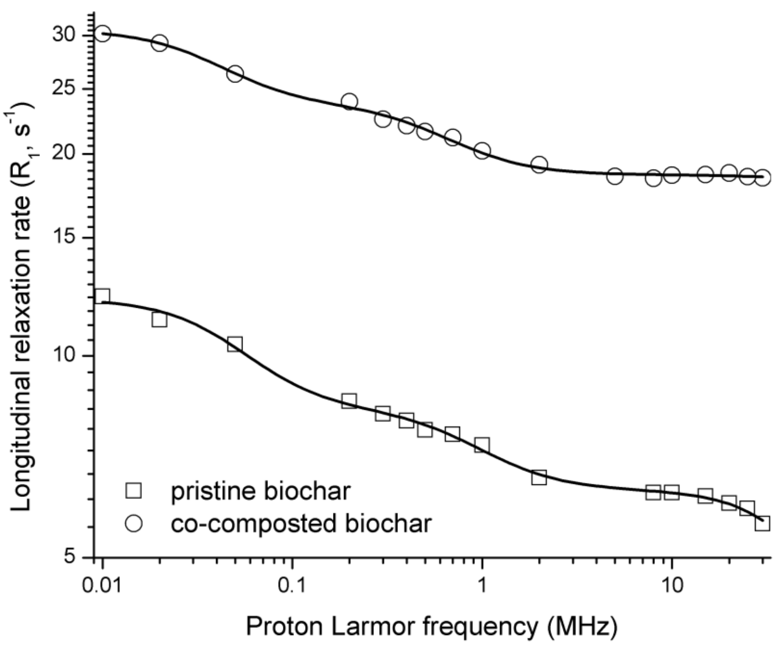

Supplementary Fig. 12: Nuclear magnetic dispersion (NMRD) profiles (i.e. the R_1=1/T_1 values vs. proton Larmor frequency) of the water-saturated pristine and co-composted biochars. In line with Fig 4 (relaxogram) the water on co-composted biochar revealed the longest R_1 values in the whole proton Larmor frequency range applied here. This can only be explained by strong interactions occurring between water molecules and the surface of the co-composted material as a consequence of the presence of H-bond donor/acceptor groups and reduced pore sizes, which means that there is less bulk water which can especially be seen in the > 1 MHz frequency area.

(A)

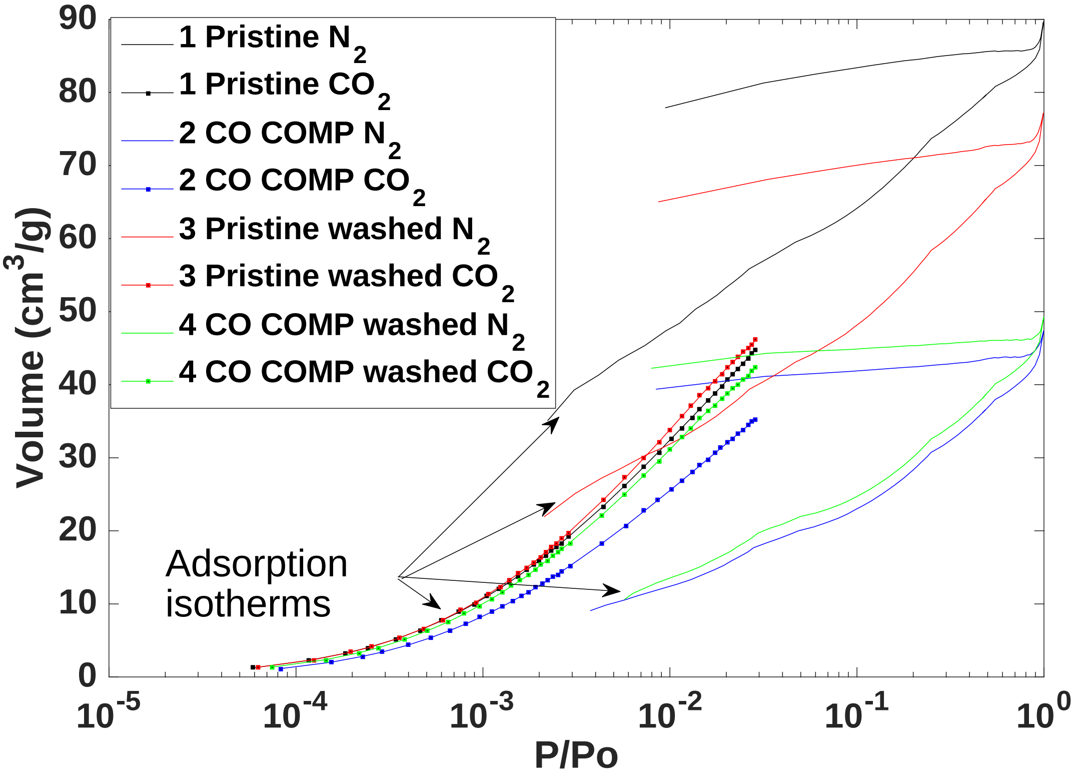
90
80
70
60
50
40
30
20
10
0
Volume (cm3/g)
1 Pristine N2
1 Pristine CO2
2 CO COMP N2
2 CO COMP CO2
3 Pristine washed N2
3 Pristine washed CO2
4 CO COMP washed N2
4 CO COMP washed CO2
Adsorption
isotherms
10-5
10-4
10-3
10-2
10-1
100
P/Po

(B)

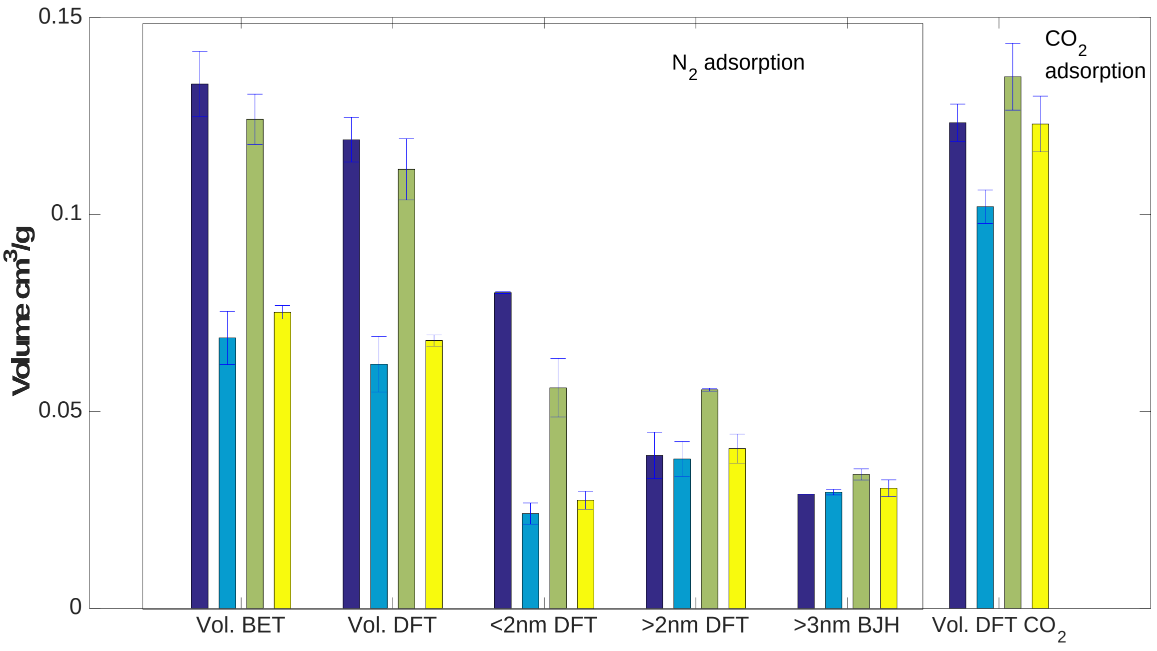
0.15
0.1
0.05
0
Volume cm3/g
N2 adsorption
CO2
adsorption
Vol. BET
Vol. DFT
<2nm DFT
>2nm DFT
>3nm BJH
Vol. DFT CO2

(C)

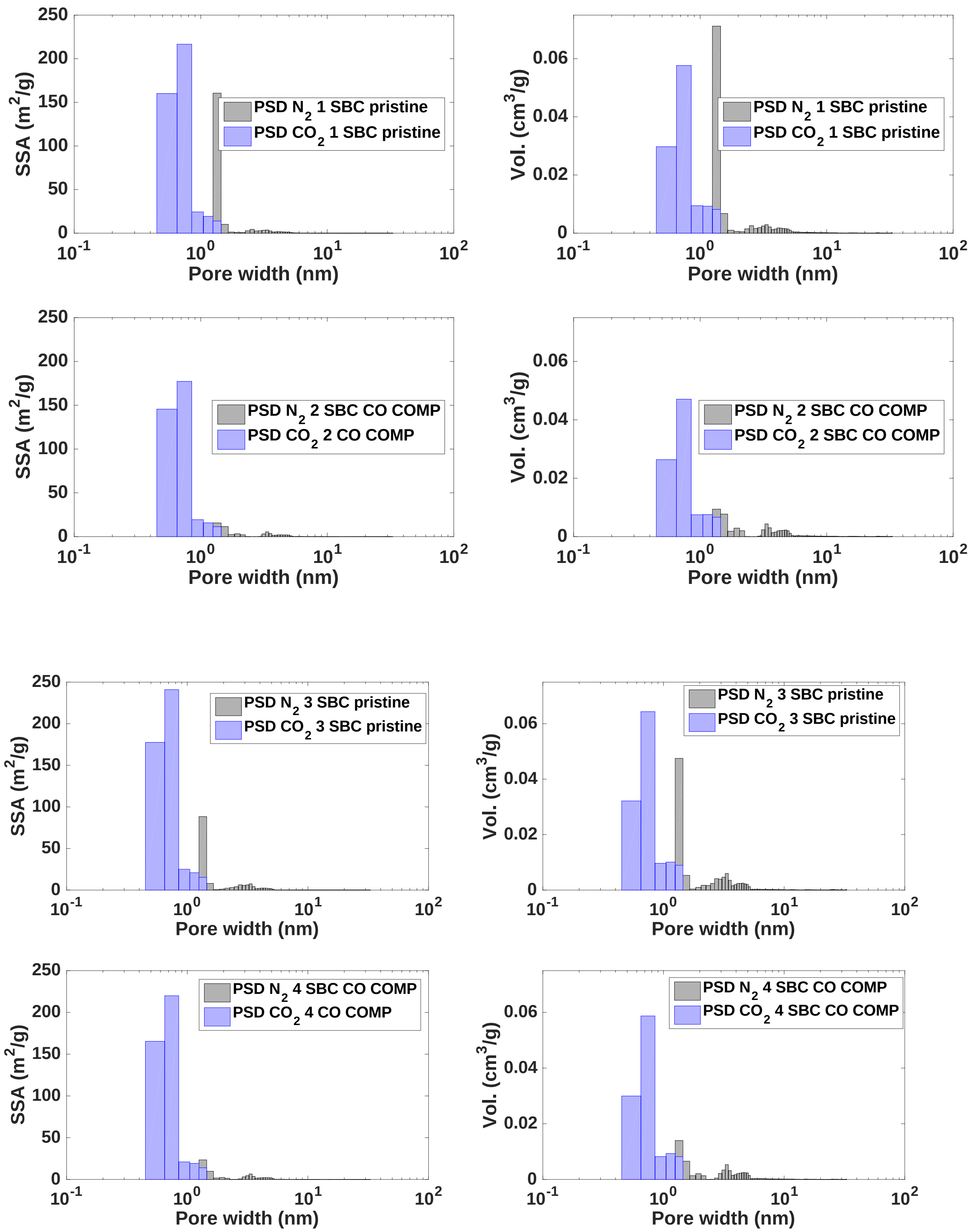

Supplementary Fig. 13: (A) Adsorption and desorption isotherms for N_2 (solid lines) and adsorption isotherms for CO_2 (dotted lines) for the four biochar samples.

(B) Total pore volume measured at a P/P_0 close to 1, total pore volume applying the QSDFT method, volume due to micropores determined with the QSDFT method, volume due to mesopores up to 34 nm with QSDFT methods and volume for pores bigger than 3 nm with BJH method. Volume due to micropores measured with CO_2 adsorption and the NLDFT method. (C) Pore size distribution for micropores and mesopores up to 30 nm in pore width according to the DFT method applied to the N_2 (grey) and CO_2 (blue) adsorption isotherms. For N_2 adsorption the QSDFT method assuming slit/cylindrical pores was employed, while for CO_2 adsorption the NLDFT was applied.

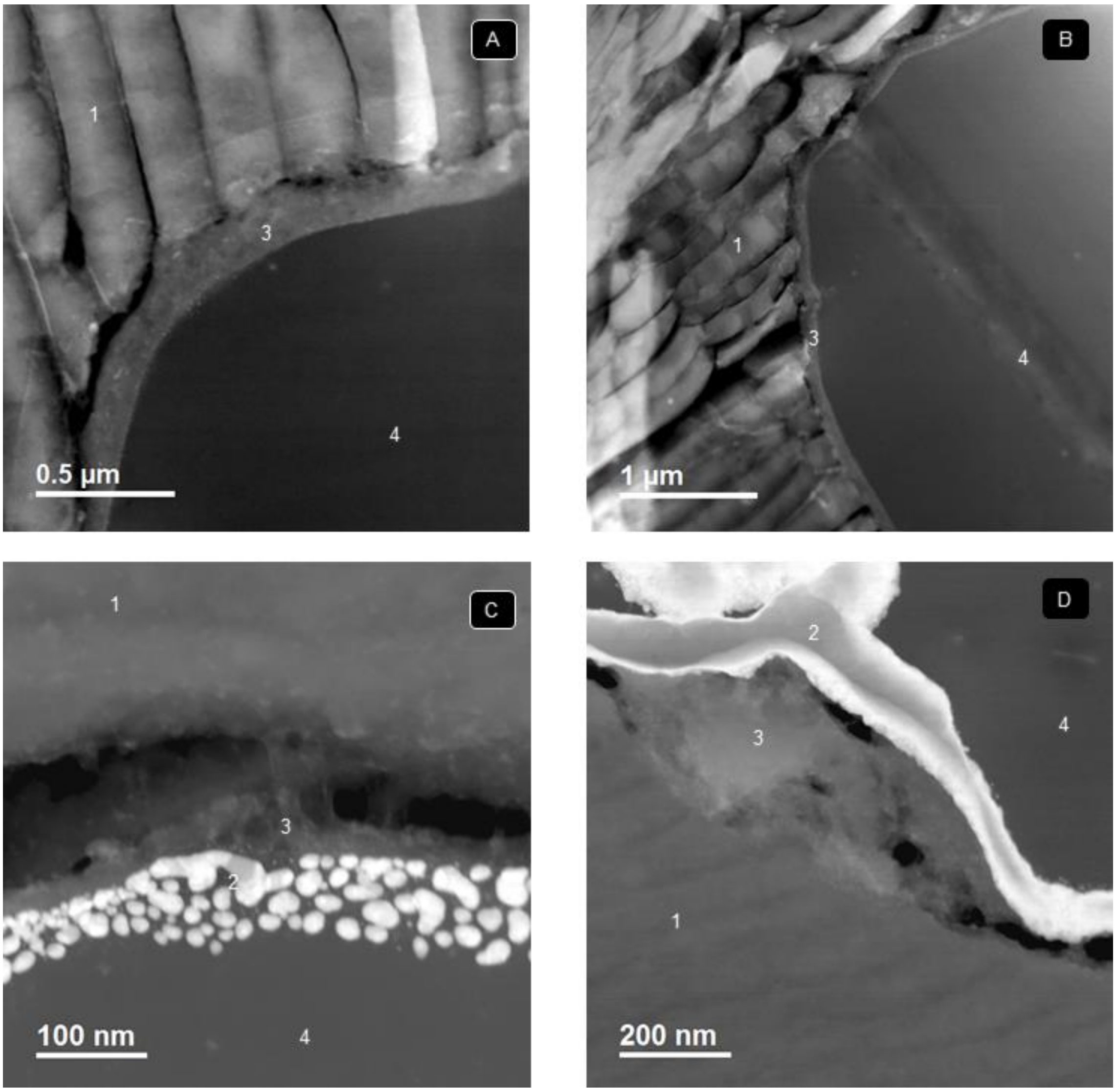

Supplementary Fig. 14: STEM HAADF (Scanning transmission electron microscopy – high angle annular dark field) micrographs and EELS (electron energy loss spectroscopy) spectra of ultra-thin sections of a co-composted biochar. A "Kon-Tiki" wood biochar [1] was co-composted in parallel to the biochar discussed in the main manuscript. All preparatory steps (compost feedstock, aeration, picking of the biochar, preparation of ultrathin sections after coating with gold) were conducted as described above. Biochar (1) shows a rippled appearance due to the ultrasonic diamond knife; Gold coating (2) is absent in (A) and (B); organic coating (3) exhibits a thickness of up to 100-200 µm; samples were embedded in epoxy resin (4). (A, B): Overview on thick coatings on pore surfaces (no gold coating visible). (C) Organic coating is shearing off due to mechanical forces between biochar and gold/resin. (D) Discrete portion of organic coating on an outer surface of biochar (thick gold coating).

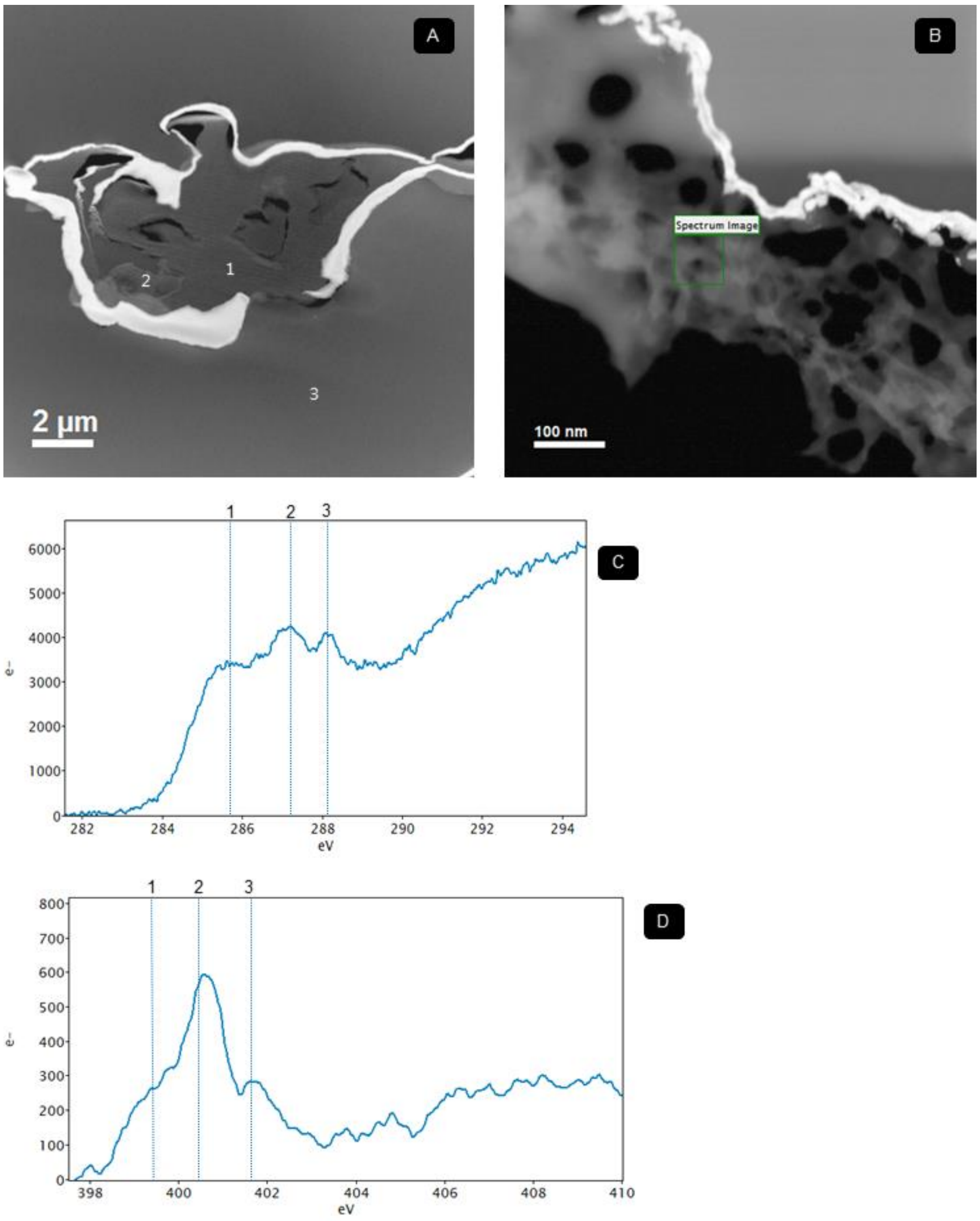

Supplementary Fig. 15: STEM HAADF (Scanning transmission electron microscopy – high angle annular dark field) micrographs and EELS (electron energy loss spectroscopy) spectra of ultra-thin sections of soil-aged biochar. (A) Overview on a small piece of biochar (1) completely embedded in epoxy resin (3) that shows a porous coating (2) of up to ~1 µm. (B) Close-up of 200-300 nm thick part of the coating showing a very heterogeneous distribution of pore sizes ranging from view to several tens of nm. (C) C-K near edge EEL spectrum obtained in a region scan located in (B). (1): 285.1 eV aromatic carbon (2): 286.2 eV, ketone C; (3): 287.1 eV, aliphatic C. (D) N-K near edge EEL spectrum obtained in a region scan located in (B). 1: 399.8 eV –> pyridinic, (2): 400.6 eV, imine N; (3): 401.8 eV, amide / peptide N [2].

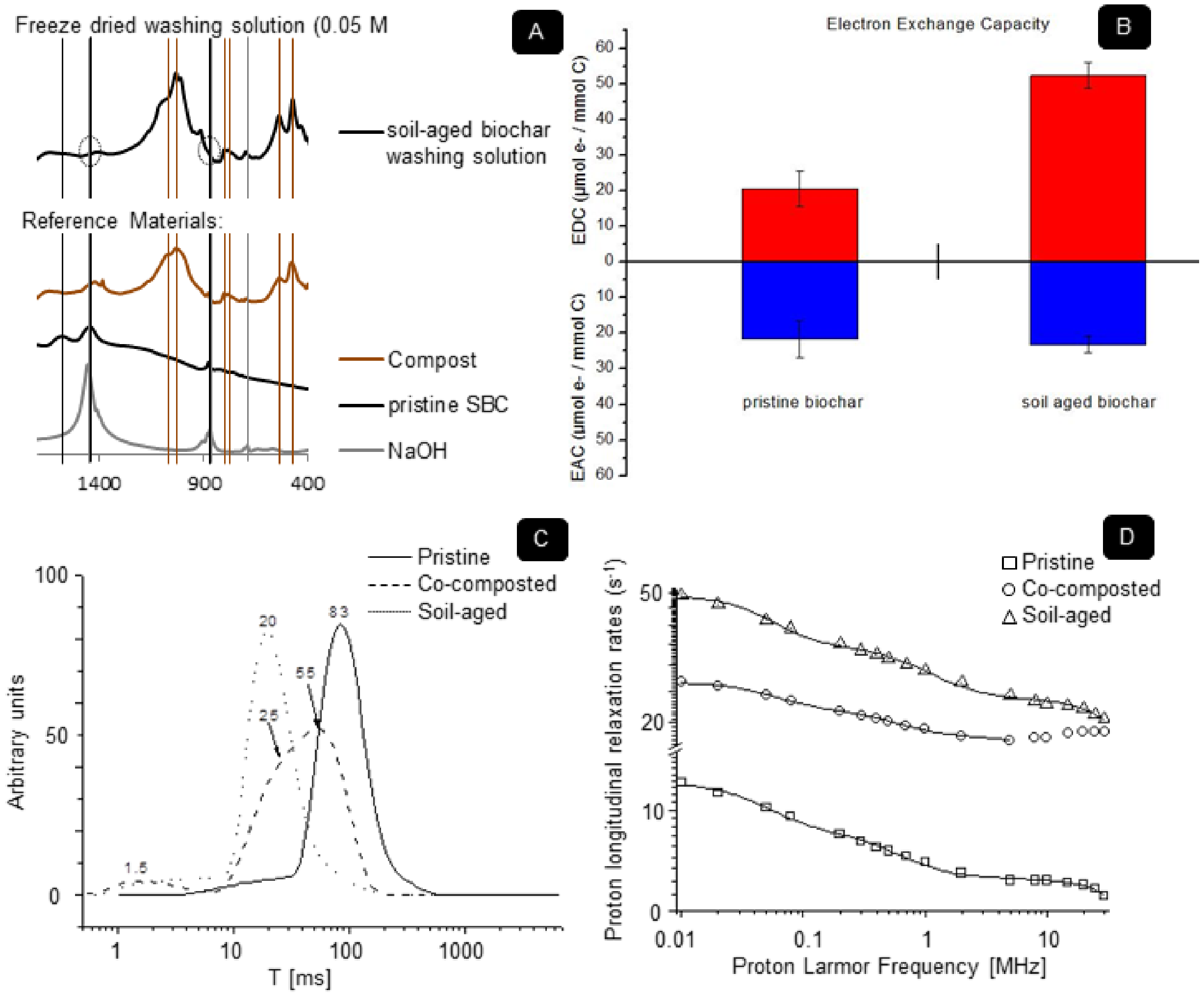

Supplementary Fig. 16: Characteristics of soil-aged biochar. (A) FTIR (Fourier-transformed infrared absorption) spectra of6freeze-dried washing solution of soil-aged biochar. Washing solution was filtered to 0.45 µm. FTIR spectra of pure compost, powdered pristine biochar and NaOH were measured as a reference. Dotted circle indicate absence of bands in soil-aged biochar washing solution that are characteristic for pristine biochar. (B) Electron accepting (blue) and donating (red) capacity (EAC/EDC) of washing eluate of soil-aged biochar and pristine biochar normalized to their carbon content. (C) Relaxograms, i.e. distribution of relaxation times according to FFC NMR relaxometry, of pristine and co-composted and soil aged biochar. (D) NRMD diagram according to (C).

(A)

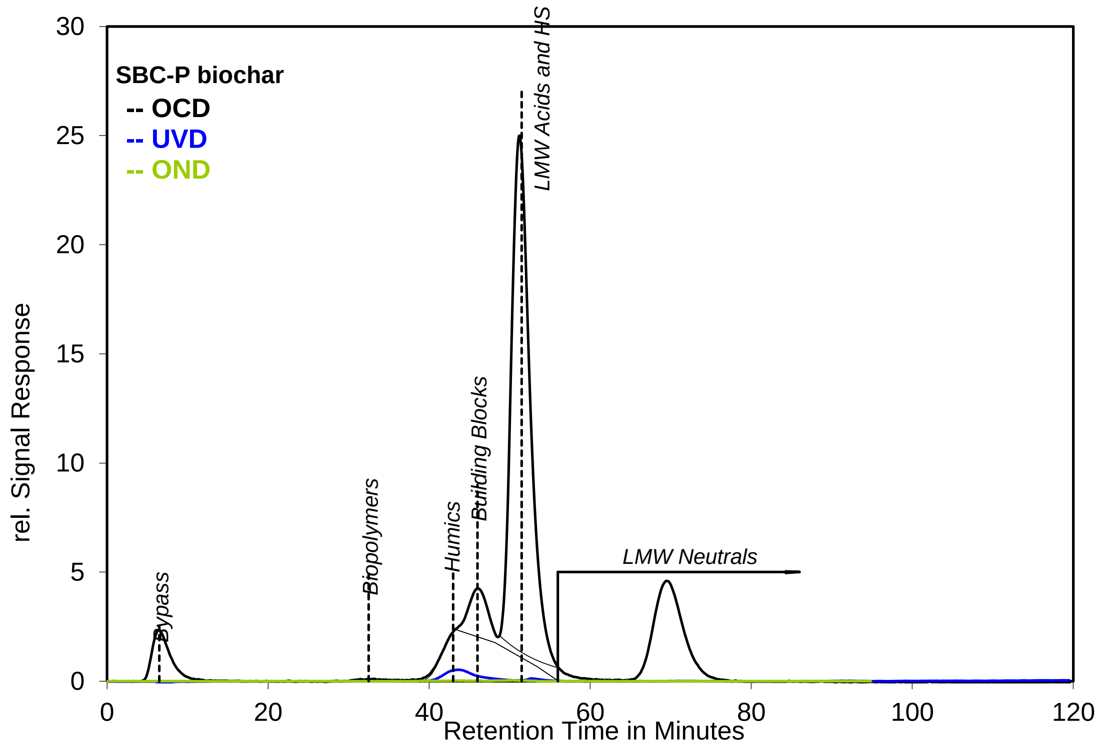

SBC-P biochar
-- OCD
-- UVD
-- OND
Bypass
Biopolymers
Humics
Building Blocks
LMW Acids and HS
LMW Neutrals
rel. Signal Response
Retention Time in Minutes

(B)

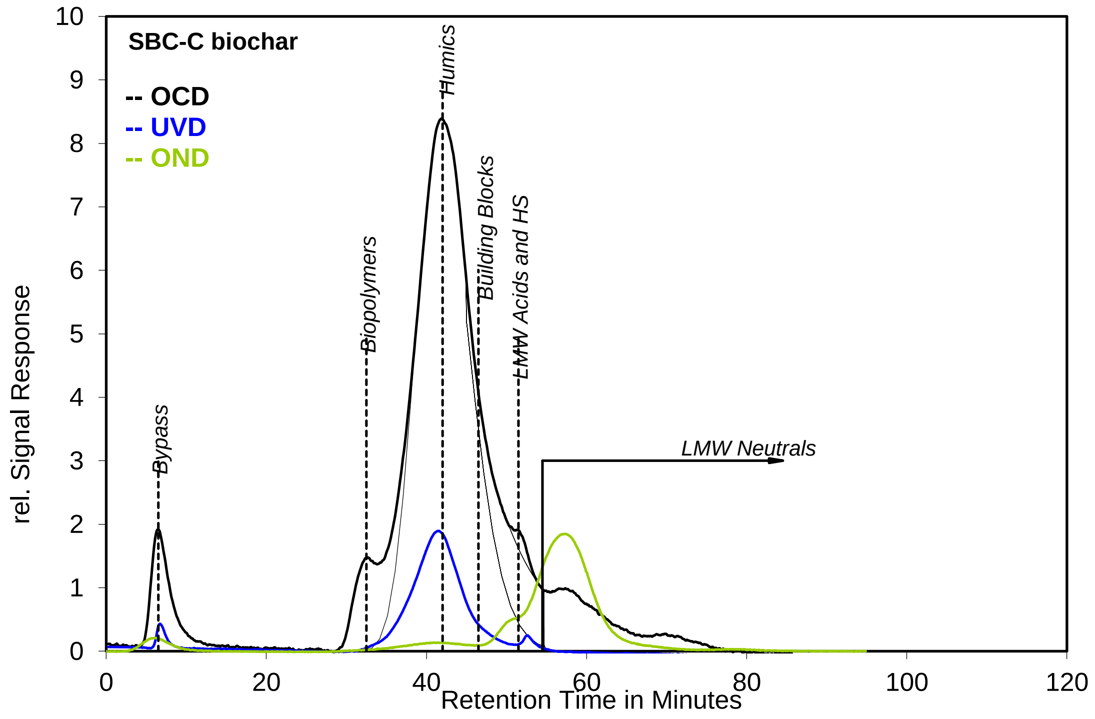

SBC-C biochar
-- OCD
-- UVD
-- OND
Bypass
Biopolymers
Humics
Building Blocks
LMW Acids and HS
LMW Neutrals
rel. Signal Response
Retention Time in Minutes

(C)

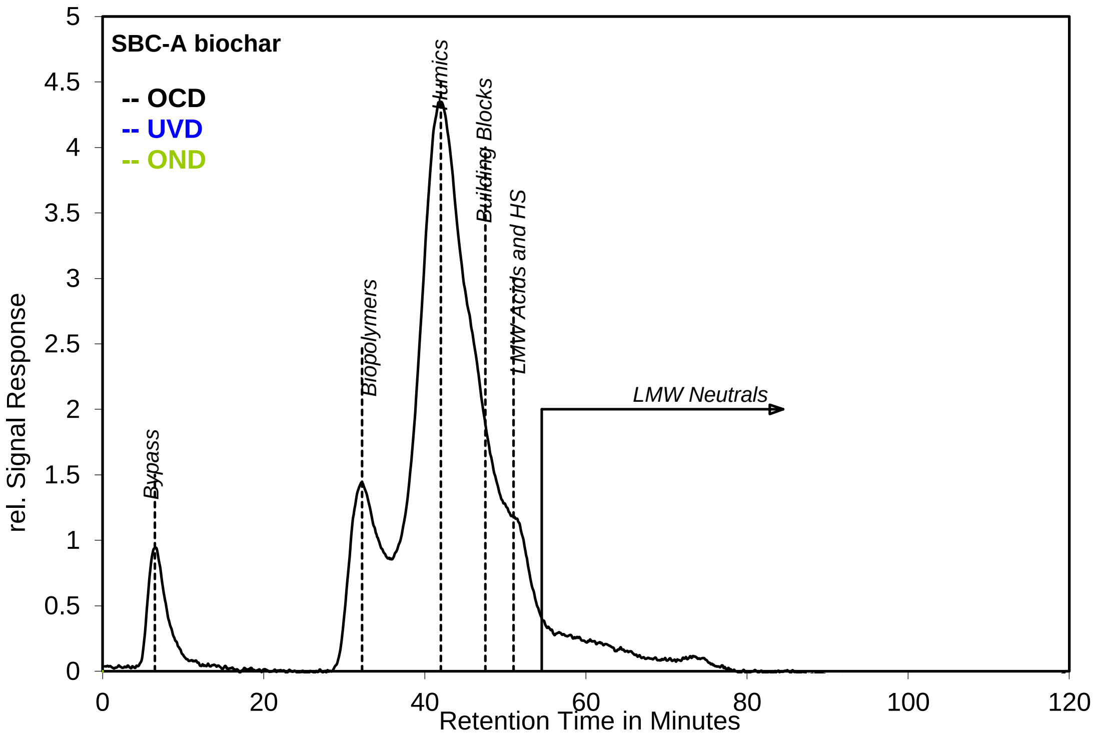

Supplementary Fig. 17: Chromatograms of LC-OCD. Chromatograms obtained from the of the water-soluble fraction of biochar (24 h extraction at 50°C): organic carbon detection (OCD), organic nitrogen detection (OND) and UV absorption at 254 nm (UVD). (A) Pristine biochar. (B) Co-composted biochar. (C) Soil-aged biochar.

Supplementary Table 1: Characterization of biochar according to the requirements of the European Biochar Certificate: Surface area according to the BET method, content of ash, salt and major elements, pH and

	BET Surface area [$m^2 g^{-1}$]	Ash 550°C [% w/w]	Element Composition [% w/w]					Molar Ratios			pH	Salt [$g\ kg^{-1}$]
			H	C	N	O	S	H/C	H/C_{org}	O/C		
EBC basic threshold				> 50				< 0.6	< 0.7	< 0.4	< 10	
Biochar 2015[1]	199.88	18.2	2.13	74.5	0.68	4.5	0.04	0.34	0.34	0.045	8.3	3.83
Biochar 2012[2]	231.85	19.4	1.03	73.2	0.64	5.7	0.06	0.17	0.17	0.058	8.0	4.3

[1] Analyzed as pristine biochar and used in the composting experiment (‘co-composted biochar’)
[2] Used in the field/soil-aging experiment (‘soil-aged‘)

Supplementary Table 2: Characterization of biochar according to the requirements of the European Biochar Certificate: Content of trace elements and polyaromatic hydrocarbons (18 EPA PAH)

	Trace Elements [mg kg^{-1}]									PAH [mg kg^{-1}][b]
	**Pb**	**Cd**	**Cu**	**Ni**	**Hg**	**Zn**	**Cr**	**B**	**Mn**	
EBC basic threshold	< 150	<1.5	< 100	< 50	< 1	< 400	< 90			12
Biochar 2015	3	< 0.2	13	5	< 0.07	52	6	24	190	4.60
Biochar 2012	< 2	< 0.2	16	8	< 0.07	45	10	36	310	6.70

Supplementary Table 1: Characterization of biochar according to the requirements of the European Biochar Certificate: Content of nutrients

	Nutrients [mg kg^{-1}]							
	**P**	**Mg**	**Ca**	**K**	**Na**	**Fe**	**Si**	**S**
EBC basic threshold								
Biochar 2015	870	2700	37000	5800	740	3900	28000	230
Biochar 2012	1400	3300	49000	8400	830	2700	22000	400

Supplementary Table 4: Chemical characterization of washing eluates normalized to dry matter of biochar. Values presented as average ± standard error.

	**Eluate of pristine biochar**	**Eluate of co-composted biochar**
Dissolved organic carbon [mg C g^{-1}]	0.03±0.00	2.47±0.09
Dissolved inorganic carbon ([bi]carbonate) [mg C g^{-1}]	0.67±0.05	1.70±0.09
Nitrate [mg NO_3^- g^{-1}]	b.d.	2.20±0.09
Potassium [mg K g^{-1}]	1.21±0.23	2.79±0.44
Magnesium [mg Mg g^{-1}]	b.d.[3]	0.02+0.00
Calcium [mg Ca g^{-1}]	0.23±0.19	0.56±0.31

[3] Below detetion limit of the ion chromatograph

Supplementary Table 5: Definitions of the different fractions of organic carbon as well as indices and their characteristics according to Huber and colleagues [3].

		Description
Fraction	Biopolymers	Organic matter with high molecular weight, including polysaccharides, proteins and aminosugars. (Molecular weight > 20 kDa)
	Humics	Mixture of acids containing carboxyl and phenolate groups produced by biodegradation of dead organic matter. (Molecular weight ~ 1000 Da)
	Building blocks	Molecular chains of polyphenolics/polyaromatic acids that have deaggregated, due to breakage of hydrogen bonding and electrostatic interactions. (Molecular weight 300 – 500 Da)
	Low molecular weight (LMW) acids	Representing protic organic acids. (Molecular weight < 350 Da)
	Low molecular weight (LMW) neutrals	Uncharged small organics, including LMW alcohols, aldehydes, ketones, sugars and LMW amino acids. (Molecular weight < 350 Da)
	Hydrophobic organic carbon (HOC)	Fraction of DOC remaining in the column, implying a strong hydrophobic interaction with the column material, comprising longer chain aliphatic and polycyclic aromatic material.
Index	Aromaticity	Aromaticity provides an estimation of the degree of aromatic and unsaturated structures of the humic fraction.
	Molecular weight	A derived value of average molecular mass of the humic fraction.
	Inorganic colloids	Negatively charged inorganic polyelectrolytes, polyhydroxides and oxyhydroxides of Fe, Al, S or Si, detected by UV light-scattering.
	SUVA	An additional parameter derived from the ratio of DOC and spectral absorption coefficient.

Supplementary Table 6: Content of total organic carbon (TOC), inorganic complexes (IC), total carbon (TC) and total nitrogen (TN) of the water-soluble fraction (24 h extraction at 50°C) according to elemental analysis.

	DOC (mg/l)	IC (mg/l)	TC (mg/l)	TN (mg/l)
**Pristine**	34.37	55.09	89.46	1.93
**Co-composted**	184.67	13.98	198.65	129.85
**Soil-aged**	19.54	21.17	40.72	6.07

Supplementary Table 7: Content of dissolved organic carbon (DOC) according to LC-OCD of the water-soluble fraction of biochar (24 h extraction at 50°C) as sum of the different fractions defined in Supplementary Table 3.

Component	Pristine mg/g C	Co-composted mg/g C	Soil-Aged mg/g C
DOC	0.5	2.14	0.22
Hydrophobic OC	n.q.	n.q.	n.q.
Hydrophilic OC	0.5	2.14	0.22
Bio-polymers	0.003	0.17	0.04
DON (bonded with bio-polymers)	0.001	n.q.	0.003
N/C	0.38		0.07
% proteins	100%		22%
Humics	0.1	1.56	0.13
DON (bonded with humics)	0.004	0.17	0.01
N/C	0.04	0.11	0.08
Aromaticity (L(mg*m)	3.21	5.46	5.16
Average Molecular Weight	809	1018	1011
Building blocks	0.04	0.19	0.04
LMW neutrals	0.09	0.21	0.01
LMW acids	0.26	n.q.	n.q.
Inorganic Colloids (m^{-1})	0.6	73.89	0.73

Supplementary Discussion

Adsorption and desorption isotherms for N_2 and adsorption isotherms for CO_2 differ strongly from a quantitative perspective. It is important to highlight that the desorption isotherms in the N_2 measurements follow almost a horizontal trajectory, i.e., all N_2 isotherms present an open loop hysteresis, which may suggest that equilibrium has not been reached, leading potentially to an underestimation of the specific surface area (SSA) and pore volume (PV). This phenomenon has been previously presented in literature [4] and is mainly caused by diffusion limitations in the pore network, which are significant when the porous structure is a complex network of mostly micropores (<2 nm), with a significant contribution of small (<1 nm) and/or constricted micropores. Such diffusion limitations are mostly observed with N_2 adsorption due to the low measurement temperature (77 K). Other phenomena such as "swelling of a nonrigid porous structure or irreversible uptake of molecules in pores (or through pore entrances) of about the same width as that of the adsorbate molecule" [5] could also contribute; these phenomena were also reported by Ravikovitch and colleagues [4].

For the above-mentioned reasons these measurements were combined with CO_2 adsorption, in order to gain more information about the porous structure. Due to the higher temperatures (273 K vs. 77 K of N_2 adsorption) and higher absolute pressures of CO_2 measurements, the diffusion of CO_2 molecules to/into the pores is much faster, even for small micropores, making it possible to characterize a part of the pore network that is potentially not being characterized by N_2 adsorption. The disadvantage of using CO_2 is that only micropores up to 1.4 nm in pore width can be measured.

This is confirmed by the results shown in Figure 8, where the SSA obtained with CO_2 adsorption (measuring pores up to 1.4 nm in pore width) is significantly higher for all biochar samples than the SSA obtained with N_2 adsorption. The results for N_2 adsorption presented in Supplementary Fig. 13B were determined applying the methods previously introduced on adsorption isotherms. Total SSA according to the BET method, total SSA, SSA due to pores <2 nm (~1 nm < pore width < 2 nm) and SSA from pores > 2 nm (2 nm < pore width < ~34 nm), according to the QSDFT method, assuming slit/cylindrical pores and SSA for pores > ~3 nm, according to the BJH method were obtained from N2 adsorption. Total SSA for pores <1.4 nm (~0.4 nm < pore width < ~1.4 nm) according to the NLDFT method was obtained from CO_2 adsorption. With respect to the pore volume, analogous explanation is valid.

Comparing these results for all biochar samples it is observed that BC_{prist} and BC_{prist}-washed have both the highest total SSA and the highest pore PV, due mainly to micropores. It is also

easy to observe that all the samples follow the same qualitative behavior, meaning that those with the highest total SSA or total PV are also those with the highest SSA or PV due to micropores and mesopores (2 nm < pore size < 50 nm). This shows that all biochar samples have similar porous network from a qualitative perspective, i.e. co-compositing leads to decrease of SSA and PV while washing increases SSA and PV, with no considerable change in the porous system, except the decrease or increase of micropores contribution to total SSA and PV, respectively. An exception to this seems to be BC_{prist}-washed. It would be expected that this biochar presents higher SSA and PV than BC_{prist} due to the washing procedure (e.g. removal of pyrogenic matter that is not part of the actual biochar matrix [6]), as for BC_{comp} and BC_{comp}-washed respectively. However, the total SSA, total PV and both SSA and PV due to micropores are lower after washing of BC_{prist}, while the SSA due to mesopores is the highest in BC_{prist}-washed among the four samples. However, looking at the results from CO_2 adsorption (Supplementary Fig. 13B), it is shown that washed BC_{prist} has the highest SSA and PV due to micropores among the four samples. This validates the previous conclusion that co-composting decreases SSA and PV and washing increases both. However, why does the pore size distribution (PSD) determined with N_2 adsorption for BC_{prist}-washed and presented in Supplementary Fig. 13C show something else, i.e. the previously explained exception? The reason may be that the N_2 based PSD results for BC_{prist}-washed are an artifact due to diffusion limitations, phenomena previously introduced, which seem to be yet stronger in this sample than in the others. According to the PSD results based on CO_2 adsorption, BC_{prist}-washed has the highest SSA and PV due to small micropores, mostly between 0.45-0.85 nm pore width. A higher contribution of these small micropores to the porous network may increase the diffusion limitations, leading to a more significant underestimation of the total SSA and SSA due to micropores. Other phenomena explaining this increase in transport limitations could be pore constriction or blocking dislocation of pyrogenic organic matter within the porous structure during the washing: pyrogenic organic matter would be removed, unveiling small micropores, as it is suggested by the CO_2 adsorption results, but part of it could remain within the porous network of the char samples, potentially constricting or blocking some connecting pores and therefore increasing diffusion limitations during N_2 adsorption. This would explain the lower value of N_2 adsorption SSA for BC_{prist}-washed in comparison to BC_{prist}. As the absolute pressure of N_2 increases in the measuring cell (higher relative pressures) the diffusion is favored, filling some of the micropores that should have been filled in advance and leading to a "false" mesoporosity or measurement artifact. Similar results about pore total or partial blocking have been already presented in literature. Pignatello et al. [7] show that pores

can be blocked by stiffness of humic macromolecules at the measurement temperatures of N_2 adsorption (77 K), while at temperatures characteristic for CO_2 adsorption (273 K) they would be much more flexible, allowing CO_2 diffusion in the pores. In the present study, the molecules (both organic and inorganic) would not be on the outer layer of the porous structure, but already inside the pores, in their way out of the biochar particle due to the washing procedure, but the impact could be similar. With respect to Supplementary Fig. 13C it is also important to clarify that SSA and PV values from N_2 adsorption of pores between 1.25 and 1.45 nm are potentially the sum of SSA and PV of these pores and also pores <1.25 nm, which due to the aforementioned N_2 limitations cannot be filled completely filled by N_2 molecules but still contribute to some extent. This explains the high values correspondent to this pore width. The QSDFT method applied in the relative pressures of the present study does not show the contribution of pores smaller than 1.25 nm.

Comparing the results further, co-composting of biochar leads to ~18 % reduction in CO_2 SSA, while to ~64 % reduction in N_2 SSA (Supplementary Fig. 13C) In the case of CO_2, this reduction takes mostly place for pores between 0.65-0.85 nm pore width. For N_2, the reduction is for the smallest pore size given by the method (1.25-1.45 nm). The reduction in total SSA is probably a combination of two phenomena: the organic matter of the coating has a lower SSA than the original biochar, hence the reduction in SSA per unit of mass of the BC_{comp}; and the organic matter may constrict some pores or even block the access, especially for N_2 adsorption, explaining the significant differences in reduction of both SSA, similar to the results reported by Pignatello et al. [7].

Washing of biochar leads to ~10% increase in CO_2 SSA for BC_{prist} and ~20% increase in BC_{comp}. In the first case, this increase was very similar in proportion for pores between 0.45-0.65 nm and pores between 0.65-0.85 nm. For co-composted biochar, the washing increased mostly the pores between 0.65-0.85 nm. This increase could be due to removal of organic matter.

With respect to their capacity of adsorbing NO_3^-, the effective radius of this ion is 0.196 nm [8], but the hydrated ion has a radius of ~0.25 nm [9], which in principle makes it still possible to be adsorbed even in biochar micropores. Therefore, an increase in SSA, including the smaller micropores (0.65-0.85 nm) could potentially improve NO_3^- adsorption. However, high surface is not enough, since the adsorption mechanism of this ion is more complex than that.

In STEM, the biochar appears as a widely homogenous matrix with a constant contrast in HAADF imaging. Micropores (<2 nm) could not be identified, although they were omnipresent according to gas adsorption measurements.

The water soluble organic component of the co-composted biochar obtained by extraction at 50°C for 24 h has significantly higher contents of both total nitrogen and carbon compared to the eluates of the fresh and soil-aged biochar (Supplementary Table 4).

According to LC-OCD, the water-soluble organic component of dissolved organic carbon (DOC) is four times greater compared to the fresh biochar and 10 times greater compared to the soil-aged biochar. LC-OCD did not detect any hydrophobic DOC (Tab. S5). The main organic molecules had a structure and composition similar to humic and fulvic acids. The concentration of the humics in the co-composted biochar was 12 times greater compared to the soil-aged sample and 15 times greater compared to the fresh sample. Quagliotto and colleagues have noted that large macro molecules (humic like substances) can act as surfactants and bind nitrates on their positive sites through electrostatic attraction [10]. The LC-OCD indicates that there is a much higher percentage of negatively changed oxihydrates in the organic layer and these could bind ammonium cations. Klučáková [11] found that nitrates can bond to solid humic particles but when these dissolve most of the nitrates would be released into solution. Carboxyl and amino groups of humic substances can bind nitrites, nitrates and ammonium depending on the pH [12].

The nitrogen content of these large molecular molecules was greater by a factor of 17 (soil-aged) and 42 (pristine). The average molecular weight and aromaticity of the eluates from the composted and from the aged sample were similar but higher than those from the fresh sample indicating a difference in the origin of these molecules. Biopolymers, building blocks and LMW neutrals where all much higher for the co-composted sample. There were no detected LMW acids for the aged and the composted sample. The major difference was the very large difference in the negatively charged inorganic polyelectrolytes, polyhydroxides and oxidhydrates of Fe, Al, S or Si, detected by UV light-scattering.

Supplementary References

1. Cornelissen G, Pandit NR, Taylor P, Pandit BH, Sparrevik M, Schmidt HP. Emissions and Char Quality of Flame-Curtain "Kon Tiki" Kilns for Farmer-Scale Charcoal/Biochar Production. *PLoS ONE* **11**, e0154617 (2016).

2. Cody GD, Gupta NS, Briggs DE, Kilcoyne A, Summons RE, Kenig F, Plotnick RE, Scott AC. Molecular signature of chitin-protein complex in Paleozoic arthropods. *Geology* **39**, 255-258 (2011).

3. Huber SA, Balz A, Abert M, Pronk W. Characterisation of aquatic humic and non-humic matter with size-exclusion chromatography – organic carbon detection – organic nitrogen detection (LC-OCD-OND). *Water Research* **45**, 879-885 (2011).

4. Ravikovitch PI, Neimark AV. Diffusion-Controlled Hysteresis. *Adsorption* **11**, 265-270 (2005).

5. Everett D, Haul L. Moscou, RA Pierotti, J. Rouquerol, and T. Siemieniewska. Reporting physisorption data for gas/solid systems with special reference to the determination of surface area and porosity. *Pure Appl Chem* **57**, 603-619 (1985).

6. Tsechansky L, Graber ER. Methodological limitations to determining acidic groups at biochar surfaces via the Boehm titration. *Carbon* **66**, 730-733 (2014).

7. Pignatello JJ, Kwon S, Lu Y. Effect of Natural Organic Substances on the Surface and Adsorptive Properties of Environmental Black Carbon (Char): Attenuation of Surface Activity by Humic and Fulvic Acids. *Environmental Science & Technology* **40**, 7757-7763 (2006).

8. Masterton W, Bolocofsky D, Lee TP. Ionic radii from scaled particle theory of the salt effect. *The Journal of Physical Chemistry* **75**, 2809-2815 (1971).

9. Richards LA, Schäfer AI, Richards BS, Corry B. The Importance of Dehydration in Determining Ion Transport in Narrow Pores. *Small* **8**, 1701-1709 (2012).

10. Quagliotto P, Viscardi G, Montoneri E, Goberro R, Adani F. Compost humic acid–like matter as surfactant. *Geophysical Research Abstracts* **7**, 10555 (2005).

11. Klučáková M. Adsorption of nitrate on humic acids studied by flow-through coulometry. *Environmental Chemistry Letters* **8**, 145-148 (2010).

12. Meinelt T, Kroupova H, Stüber A, Rennert B, Wienke A, Steinberg CE. Can dissolved aquatic humic substances reduce the toxicity of ammonia and nitrite in recirculating aquaculture systems? *Aquaculture* **306**, 378-383 (2010).
