# Supplementary material for: Organic coating on biochar explains its nutrient retention and stimulation of soil fertility
Source: Nat Commun. 2017 Oct 20;8:1089. doi: 10.1038/s41467-017-01123-0 (PMC5715018; doi:10.1038/s41467-017-01123-0)
Supplement: Supplementary file 2 — Peer Review File [file 41467_2017_1123_MOESM2_ESM.pdf]

Reviewers' Comments:

Reviewer #1:
Remarks to the Author:
This work has performed a thorough molecular level study on the mechanism of improved soil quality of biochar amended soil, by combining use of various spectroscopy and mass spectrometry techniques. Heterogeneously accumulated organic matters on the biochar surfaces were attributed to the origin of the high nutrient of co-composed biochar. This is a well described study that deserves the publication.

I just have one minor comment about the interpretation of APPI FTICR data set in page 6, line 115-116: "... co-composting increased both N/C and O/C ratios in compounds on the surface of biochar that desorb at atmospheric pressure (Fig 2a, Fig S3)."
This may not be exactly correct interpretation of the data set and might mislead the readers as is written. There is no direct evidence for the overall increase of all O/C ratio in this data set but maybe only N/C. What is clear in this data set is, the increase of CHON class compounds and the decrease of relative composition of CHO class (Fig. 2a). It is true though O/C might increase within CHON class (Fig S3). I would suggest to revise this sentence to avoid the confusion of the readers.

Reviewer #2:
Remarks to the Author:
This manuscript presents a detailed molecular investigation of the morphology, composition and functionality of biochar surfaces. The study is well designed and the analyses expertly conducted. The main finding is that co-composting with organic matter, which is known to increase the biochar's potential as a slow-release nutrient reservoir, creates organic coatings on biochar surfaces. These coatings are then shown to provide the functionality critical for nutrient retention and release, a function previously assigned to oxidized biochar surfaces. I believe this insight will be of interest to the agronomy and environmental science community.

My main concerns are the following:
1) The authors argue that organic coatings, rather than surface oxidation, provides functions related to nutrient retention. However, it equally possible that both act in concert. The pristine biochar surfaces may have to be oxidized first to enable and enhanced sorption of organic compounds and nutrients that constitute the coating described here. I recommend moving away from this absolute "oxidation versus coating" argument. Instead, it may be worth offering the interaction between surface oxidation and sorptive coating as an intriguing new research question that logically follows from this work.
2) Some statements related to biochar stability appear far-fetched (L94 and L181-182). The long-term stability of these biochars was not experimentally examined. is not really the focus of this paper and statements to this effect seem misplaced and unnecessary.
3) The authors' line of reasoning relies heavily on data in the SI. I recommend moving critical data into the main manuscript body. Or, if space in the main manuscript is limited, make figures in the SI more readily comprehensible.

SPECIFIC COMMENTS

L53-55: I wouldn't pose this as an either or question. In fact, I would think that surface oxidation provides the reactive surface sites needed for sorptive coatings to form.

The first two paragraphs of the introduction are a long laundry list of biochar uses and properties. The introduction could be more captivating for a high impact journal such as Nature Communications.

L65ff: While I agree that many mechanistic questions remain, it's not fair to say there is no work trying to address it:

Ajayi AE, Holthusen D, Horn R (2016) Changes in microstructural behaviour and hydraulic functions of biochar amended soils. Soil and Tillage Research 155:166–175. doi: 10.1016/j.still.2015.08.007

Conte P, Laudicina VA (2017) Mechanisms of Organic Coating on the Surface of a Poplar Biochar. Current Organic Chemistry 21:1–7.

Mukherjee A, Zimmerman AR, Hamdan R, Cooper WT (2014) Physicochemical changes in pyrogenic organic matter (biochar) after 15 months of field aging. Solid Earth 5:693–704. doi: 10.5194/se-5-693-2014

Wiedner K, Fischer D, Walther S, et al (2015) Acceleration of Biochar Surface Oxidation during Composting? J Agric Food Chem 63:3830–3837. doi: 10.1021/acs.jafc.5b00846

L116: It appears that the N-rich coating as evidenced by X-ray microscopy is very important for the authors' argument. Yet these data are buried deep in the supporting info. Moreover, the authors only refer to Fig. S-4c, while the rest of the figure is not discussed. It is difficult, among all the other panels, to get the main point of this and many other figures in the SI.

Reviewer #3:
Remarks to the Author:
This manuscript aims to address the difficulty biochar scientists have had in the past to explain the differences between the expected improvements in soil fertility based on studies of the terra preta and the apparently inert nature of biochar upon exiting a pyrolyzer. The suggestion here is that co-composting, that is oxidative decomposition of organic matter in the close proximity of the biochar, is the cause of the properties of the biochar particles resulting in such high soil fertility. From a historical perspecitive, this hypothesis is plausible since char was almost always found in locations with other organic wastes (midden heaps). Work in the last decade showing char surface oxidation has so far been unsatisfactory to completely explain the changes of biochar "aging". While literature does contain studies about char-mineral interactions, this manuscript is one of the first to use methods capable of characterizing the char organic-compost organic-compost inorganic interactions at the char surface. The authors' conclusion that biochars should be mixed wtih organic matter, preferably in a co-composting scenario, prior to soil application for more impactful soil fertility short-term changes is reasonable and well supported by the coating property evidence. This manuscript can serve as a list of potential evaluation methods for future co-composting/biochar field studies to determine how frequently there coating phenomena can be correlated with soil-plant relationships; as such, publication will be very timely and helpful to researchers in the field now, many of whom are working on the nutrient aspects of biochar soil amendments to address economic issues.

For the most part, the study methods are thoroughly described and cited. Two concerns with the described methods are the way that biochar particles were "picked manually" and the use of washing in some of the characterization method sample preparation. How many char particles were selected? Were those particles mixed/homogenized before the various extractions/characterizations? On how many particles were these coatings observed? Were any of the particles not coated? Were any measurements taken of the microbial activities/DNA content of these coatings to estimate what portion of the coating was microbial biomass and what part was abiotic/humus? For the washing, why was the washing required? How did the char coatings change with washing vs. non-washing?

****

Responses to Reviewer #1:

1) *I just have one minor comment about the interpretation of APPI FTICR data set in page 6, line 115-116: "... co-composting increased both N/C and O/C ratios in compounds on the surface of biochar that desorb at atmospheric pressure (Fig 2a, Fig S3)." This may not be exactly correct interpretation of the data set and might mislead the readers as is written. There is no direct evidence for the overall increase of all O/C ratio in this data set but maybe only N/C. What is clear in this data set is, the increase of CHON class compounds and the decrease of relative composition of CHO class (Fig. 2a). It is true though O/C might increase within CHON class (Fig S3). I would suggest to revise this sentence to avoid the confusion of the readers.*

We agree with the reviewer and have revised the manuscript accordingly (L123-129; line numbers refer to revised manuscript with changes accepted). We specifically now discuss the N/C and O/C ratios.

****

Responses to Reviewer #2:

1) *The authors argue that organic coatings, rather than surface oxidation, provides functions related to nutrient retention. However, it equally possible that both act in concert. The pristine biochar surfaces may have to be oxidized first to enable and enhanced sorption of organic compounds and nutrients that constitute the coating described here. I recommend moving away from this absolute "oxidation versus coating" argument. Instead, it may be worth offering the interaction between surface oxidation and sorptive coating as an intriguing new research question that logically follows from this work.*

We appreciate this excellent suggestion and have included this line of thought to the discussion of the manuscript. As suggested, we also tried to reduce the "sorption vs. oxidation" point of view on the level of ageing processes (although we would like to highlight that, when looking at biochar functioning, our data provides evidence for a superior role of sorptive coating compared to surface oxidation).

2) *Some statements related to biochar stability appear far-fetched (L94 and L181-182). The long-term stability of these biochars was not experimentally examined. is not really the focus of this paper and statements to this effect seem misplaced and unnecessary.*

We agree with the reviewer, that we actually do not provide any direct data on the long-term stability of the biochars investigated here. However, we show that the "backbone" of biochar was not altered chemically, which we look at as a strong indicator for biochar stability. Additionally, there is still a debate on extraordinary long-term stability of many biochars and the concept of the organic coating might contribute as a (partial) explanation for biochar stability. Nevertheless, we followed the reviewer's suggestion and toned down our statement (L. 200-202; revised manuscript with changes accepted).

3) *The authors' line of reasoning relies heavily on data in the SI. I recommend moving critical data into the main manuscript body. Or, if space in the main manuscript is limited, make figures in the SI more readily comprehensible.*

We agree with the reviewer and moved critical data to the main manuscript:

- Nitrate slow release (Fig 1)
- Parts of the data on Scanning Transmission X-ray Microscopy (Fig 4)
- Specific surface area (Fig 8)

4) *L53-55: I wouldn't pose this as an either or question. In fact, I would think that surface oxidation provides the reactive surface sites needed for sorptive coatings to form.*

In general, we agree and appreciate the reviewer's view that the sorption of organic matter could depend on biochar surface oxidation to create the sites for sorption. We include this idea based on his suggestion into the MS in the discussion of the origin of the coating. However, independent of the origin of the coating, the "*functioning of biochar in soil*" (Lines 52-54; revised manuscript with changes accepted) will be still determined by the properties of the organic coating. Therefore, we prefer not to change the abstract.

5) *The first two paragraphs of the introduction are a long laundry list of biochar uses and properties. The introduction could be more captivating for a high impact journal such as Nature Communications.*

As recommended we have revised and streamlined the introduction.

6) *L65ff: While I agree that many mechanistic questions remain, it's not fair to say there is no work trying to address it:*

- *Ajayi AE, Holthusen D, Horn R (2016) Changes in microstructural behaviour and hydraulic functions of biochar amended soils. Soil and Tillage Research 155:166–175. doi: 10.1016/j.still.2015.08.007*
- *Conte P, Laudicina VA (2017) Mechanisms of Organic Coating on the Surface of a Poplar Biochar. Current Organic Chemistry 21:1–7.*
- *Mukherjee A, Zimmerman AR, Hamdan R, Cooper WT (2014) Physicochemical changes in pyrogenic organic matter (biochar) after 15 months of field aging. Solid Earth 5:693–704. doi: 10.5194/se-5-693-2014*
- *Wiedner K, Fischer D, Walther S, et al (2015) Acceleration of Biochar Surface Oxidation during Composting? J Agric Food Chem 63:3830–3837. doi: 10.1021/acs.jafc.5b00846*

We thank the reviewer for suggesting these references. The reference Wiedner *et al.* was already included in our MS, all other references are now added to the introduction; Conte and Laudicina (2017) also added important arguments to the discussion of our results.

7) *L116: It appears that the N-rich coating as evidenced by X-ray microscopy is very important for the authors' argument. Yet these data are buried deep in the supporting info. Moreover, the authors only refer to Fig. S-4c, while the rest of the figure is not discussed. It is difficult, among all the other panels, to get the main point of this and many other figures in the SI.*

We agree and have moved parts of Fig S4 (b, c, d) to the MS as a new Fig 4.

****

Responses to Reviewer #3:

1) *How many char particles were selected? Were those particles mixed/homogenized before the various extractions/characterizations?*

We would like to thank the reviewer for bringing up these important methodological questions. As suggested, we have added important information on the sample selection and treatment. For example, we now explain in the methods section: "50 kg of biochar-amended compost were stored for analysis in a plastic bag. For each analytical procedure (extraction, characterization), a subsample of compost was collected as a composite sample from this bag. 10 to 30 biochar particles were separated from this individual subsample and then prepared for analysis." We furthermore now state in the methods section that "The actual number of biochar particles finally involved in analysis varied strongly between the techniques. Due to limited instrument time (for example limited beamtime at the synchrotron), e.g. only 3 particles of each treatment could by analyzed by STEM and 2 for STXM, while extraction or powder-based analysis (gas adsorption) where based on at least 20 particles".

We would also like to mention: As storage of biochar is always problematic to avoid any storage-related changes (oxidation, drying, …), we decided to leave the biochar in the biochar-amended compost as long as possible, thus we always homogenized the compost prior to subsampling, but could not homogenize or mix the actual biochar particles used in the study as a whole.

2) *On how many particles were these coatings observed? Were any of the particles not coated?*

We found evidence for the organic coating on all particles investigated, this is now explicitly mentioned in the beginning of the discussion. However, we also want to highlight that the coating does not cover the particles completely. This important point is included also into the discussion.

3) *Were any measurements taken of the microbial activities/DNA content of these coatings to estimate what portion of the coating was microbial biomass and what part was abiotic/humus?*

Unfortunately, we do not have data on the microbial activity or DNA content of the coating. We agree with the reviewer that microbial biomass could contribute to the formation of the coating, as it is widely accepted in the literature, that biochar is a habitat for microbes. However, LC-OCD revealed that most organic molecules extracted with water at 50°C for 24 h had a structure and composition similar to humic and fulvic acids. We added this important point to the discussion.

4) *For the washing, why was the washing required? How did the char coatings change with washing vs. non-washing?*

The washing was performed to test the hypothesis that the formation of the coating is reversible, i.e. the coating can be completely removed by a washing procedure. This was not the case, as we showed in Fig S9; a granular material formed on the surface of co-composted biochar as a consequence of the washing. However, a sufficient amount of the coating was dissolved and/or suspended in the washing procedure and could be analyzed (Fig. 6).

Reviewers' Comments:

Reviewer #1:
Remarks to the Author:
The authors properly addressed my comments.

Reviewer #2:
Remarks to the Author:
My previous concerns have been addressed in full.

Reviewer #3:
Remarks to the Author:
The authors have adequately addressed the issued raised in the first review, specifically the clarification of organic coating vs. surface oxidation, addition of methodology details, and selection of figures to include in the main manuscript. The manuscript could be acceptable with the two edits below:

The caption to the images in Figure 5 is somewhat challenging to understand. Are the three images from different positions on the same biochar particle or different particles? Perhaps use of different colors for the indicators would help or separation of the figure into three figures to clarify that the figures are paired. On the first glance, this reviewer mentally combined all blue boxes/spectral features, etc. Some clarification would be appreciated.
Figure 8 requires a key to show which color of bar corresponds to which sample.

Reviewer #3 (Remarks to the Author):

The authors have adequately addressed the issued raised in the first review, specifically the clarification of organic coating vs. surface oxidation, addition of methodology details, and selection of figures to include in the main manuscript. The manuscript could be acceptable with the two edits below:

The caption to the images in Figure 5 is somewhat challenging to understand. Are the three images from different positions on the same biochar particle or different particles? Perhaps use of different colors for the indicators would help or separation of the figure into three figures to clarify that the figures are paired. On the first glance, this reviewer mentally combined all blue boxes/spectral features, etc. Some clarification would be appreciated.
Figure 8 requires a key to show which color of bar corresponds to which sample.

Authors' response:

We appreciate the suggestions of the reviewer and added a sentence to the caption of Figure 5 and revised the figure to ease the reading of the figure. We also revised the caption of Figure 8. Figure 8 contains a key to the color code of the samples.
